# The Life of Edward Jenner, M.D. &c. &c.

**Published:** 1838-10-01

**Authors:** 


The Life of Edward Jenner, M.D., LL.D., F.R.S., &c. &c., with Illustrations of his Doctrines, and Selections from his Correspondence. By *John Baron*, M.D., F.R.S., &c., &c. In two Volumes, 8vo. Colburn, London, 1838.

Of all species of composition, biography, perhaps, best combines amusement with instruction. It seems to annihilate space and time, and makes us the companions and the confidantes of the illustrious dead. Stripped of the sock, the buskin, and the mask, in which they have been disguised on the stage of history, biography exhibits them behind the curtain, attired like ordinary men, and surrounded with all the common-places of humanity. We cannot wonder that our curiosity should be vividly excited, to learn how beings, who appear through the mist of years and from the elevation of their fame, so superior to ourselves, behaved, at the social board or the family circle, as the neighbour, the husband, or the father.

Did biography, however, go no farther than this, we should hesitate to admit its claims to furnishing a high order of instruction. It would gratify an amiable curiosity, and do little more. It is when conspicuous merit is made familiar, and by its familiarity is rendered a practicable object of imitation, that biography grows really useful. Its highest and its legitimate office is to paint the progress of industry, the rewards of genius, the necessity for good conduct, and the effects of bad. The virtues of one man are thus made to prompt posterity to excellence, and the moral lesson has the double advantage of doctrine and of illustration, of abstract truth, and of positive matter of fact.

The life of a man who has benefited humanity, by discoveries in science, is the best commentary on the history of science itself. It shews the gradual manner in which truth has been detected or confirmed, and making us acquainted with its slow though steady advance, teaches us to persevere in the face of difficulties and amidst apparent defeats. Can any lesson be more noble, can any be more valuable than this? Biography gives an immortality to virtue, due to it as a reward, politicly presented to it as an example; its praises become a cheap retaining fee, given by the world to the good and great, for securing to itself their services.

These reflections were excited, by a glance at the biography of Jenner. We see in him the combination of the virtuous and the scientific benefactor of his species, and while he forms an epoch in the history of the world, generations yet unborn and unprotected by his knowledge, will learn that the man to whom they owe their lives, was unblemished in his own—and that while his science will give them the physical benefits of existence, his example can teach them how to render that existence a moral and a social blessing. In short the history of Jenner is the history of a good man, of a great discoverer, of an ornament to our profession, and of one of the causes which have elevated us from the low rank of disputatious empirics, in which we had for centuries been lost. It is a duty to the profession to make such a history known, to hold up such an example, to seize the opportunity of confirming the noble sentiments of the old amongst us, and of instilling them into the young.

Dr. Baron is well qualified from reputation and talent to be the biographer of Jenner. Our readers will perceive that he is also qualified by opportunities.

"This work has been composed from materials of the most authentic description, the whole of the notes and correspondence of Dr. Jenner having been put into my hands by his executors. My close and unreserved intercourse with him, and my intimate knowledge of his sentiments and habits of thinking on all subjects during the last fifteen years of his life, probably induced them to believe that I was not an unfit person to draw from such sources an accurate delineation of his character and opinions.

Many reasons, with which I need not trouble the public, would have induced me to shrink from the labours of such a work; and nothing certainly could have reconciled me to the attempt had I not been influenced by the most sincere veneration for the name of Jenner, and by the conviction that the confidence with which he honoured me *did* afford me facilities for acquiring an insight into his feelings and motives, by which I have been enabled to speak without hesitation or doubt on all those points that most concerned either his conduct as a man, or the nature of his doctrines.

Notwithstanding these encouragements, I cannot but own that I have entered upon this undertaking with a degree of anxiety in which I can scarcely expect any to sympathize. I trust that I am not deceiving myself when I say that nothing of a personal nature prompts this avowal. It is of moment that a true and faithful portrait should be drawn of so distinguished an individual; that those, who have admired and extolled him as a great benefactor of our race, may know that on many other grounds he was worthy of their highest regard and warmest affection. It cannot be expected that there should be an uniformity of sentiment on momentous questions of a professional or scientific nature, but I shall ever have cause to lament if, through any defects of mine, the kindness, the rectitude, the consistency, and the unextinguishable ardour and devotedness of Jenner in a glorious cause do not shine conspicuous in every act of his long and laborious life.

On a knowledge of these things my pretensions as his biographer chiefly rest. If I have failed in imparting that knowledge I shall have a cause for regret which no deficiency in any other part of my design could occasion. The world at large has felt and acknowledged the blessings of his great discovery; but few are aware how numerous were his claims to admiration." *Introd.* xi.

"The private history of Jenner and of his labours could only be fully derived through those channels of information which have been open to me. I have selected such facts as are for the most part new, I believe, to the public. In order to authenticate the narrative, and to impart to it that spirit which original documents alone can give, I have embodied such as appeared to me most interesting, in the text. I have preferred this method either to that of printing them as an appendix, or in the form of notes. One of the chief reasons for this decision arose from the nature of the transactions that I was called on to record. Many of them regarded the conduct of individuals; and I saw no method of escaping charges of partiality or unfairness except by bringing forward proofs that cannot be denied, and which will show that I have dealt honestly by all. In a question which has in a peculiar degree excited strong feelings, both with the public and in the profession, I can scarcely hope to have written, on all occasions, so as not to have called up recollections of an unpleasant kind in the minds of some. However this may be, I trust it will be apparent that truth and moderation have guided me throughout the whole work." xix.

Dr. Baron observes a little farther on :—

"For almost all his early letters I am indebted to his friend the late Edward Gardner, of Framptom-upon-Severn, who bequeathed them to me on his death-bed. These, together with those addressed to myself, form a series which touches on almost every subject of interest, whether of a public or domestic nature, during the last forty years of his life.

I have also had letters and extracts of letters transmitted to me by Colonel Berkeley, Thomas Paytherus, Esq., Henry Hicks, Esq., James Carrick Moore, Esq., Charles Murray, Esq., and Henry Jones Shrapnell, Esq., to all of whom I beg to return my thanks.

Animated as I have been by the most ardent and devoted attachment to the memory of Jenner, it cannot be expected that I should either repress my feelings, or employ cold and measured language to mark my sense of the value of his labours, and the importance of their results. I can scarcely expect that my reader will go along with me on all such occasions, but I *do* indulge the hope that he will see reason to forgive that warmth in which he may not be able to participate. Jenner's nature was mild, unobtrusive, unambitious; and many who have done justice to his discovery have still to learn how beautifully the singleness of his heart and his genuine modesty graced and adorned that splendid reputation which the wonderful consequences of his labours had acquired for him. In every private affair, in every public transaction one principle guided him. The purity of his motives and the disinterestedness of his actions have, by no means, yet been duly acknowledged: had those who opposed him and vaccination known how little of selfishness, of vanity or of pride entered into his character, they would, I am persuaded, deeply lament the wounds which they inflicted; and in the place of bitterness and reproach would have found cause for unmixed esteem and approbation." xxi.

Our readers must already perceive that the biographer of Jenner has all the warmth of a friend, and that that illustrious man will receive no sullen nor niggard meed of approbation at his hands. Nor should he at the hands of any man, for his life was devoted to carrying his discovery into efficient operation, and even Howard did not excel him in genuine philanthropy.

In the history of Jenner there are two subjects to attract attention. They are neither strictly separable, nor inextricably entangled. The one is the life of the man—the other the nature of vaccination, its present state, and its future prospects.

The life of Jenner is, in a great measure, the history of vaccination, it is true. But it is also the history of discovery in general. In it we shall see the force of prejudice, the obstinacy of error, the spitefulness of rivalry; we shall see a good and a great man struggling through bad report to uphold and to disseminate the truth, borne up against the tide of authority, ridicule, calumny, and what is hardest of all to overcome, indifference, by the consciousness of being the bearer of a noble though a contemned boon to his species; we shall see this man triumphant at last, thanks to the truth and to his own perseverance; we shall see him hailed not merely as the saviour of his country but of the human race; and his victory will read a lesson to industry and genius, that will tell them to disregard obstacles and to laugh at difficulties in the pursuit of noble ends by honest means. The life, then, of Jenner ought to be familiar as household words with our young men.

The scientific history of vaccination, and the questions that depend upon it, are of no slight interest at the present moment. Small-pox has lately appeared in a general, if not in an aggravated form, and vaccination has, unfortunately, failed, in many cases, to offer the security that had been expected from it. Under such circumstances, it becomes an object of the first importance to be intimately acquainted with the nature and properties of vaccination, in order that we may determine what its protective powers are, what portion of its failure is attributable to an inherent imperfection in those powers, and what to an improper mode of performing the process itself.

It would be impossible, in one article, to do justice to the biography and the discovery of Jenner. We shall content ourselves at present with the former, and reserve the examination of the latter to a future, though an early opportunity. In the present article we shall trace the history, and exhibit the most interesting circumstances connected with the life of the man, and we are sure it will neither be an useless nor an unpleasing office to exhibit his career.

Edward Jenner was born in the vicarage at Berkeley, in Gloucestershire, on the 17th of May, 1749. He was the third son of the Reverend Stephen Jenner, A.M. of the University of Oxford, Rector of Rockhampton, and Vicar of Berkeley. His mother was the daughter of the Rev. Henry Head, of an ancient and respectable family in Berkshire. This clergyman once held the living of Berkeley, and had, at the same time, a prebendal stall in the Cathedral of Bristol.

Besides his church-preferments, the father of Jenner possessed considerable landed property, the family being of great antiquity in Gloucestershire and the neighbouring county of Worcester. It has produced several eminent men, among whom may be mentioned Dr. Thomas Jenner, President of Magdalen College, Oxford, the immediate predecessor of the pious and learned Dr. George Horne. Jenner's father had been tutor to a former Earl of Berkeley; and the late Earl, his brother the admiral, and, indeed, the whole of that noble house, always evinced a very strong regard to him and to his family. This excellent and devout man was cut off not long after the birth of his son Edward, at the age of 52, in the year 1754. This heavy loss was as much as possible alleviated by the affectionate care and judicious guidance of his eldest brother, the Rev. Stephen Jenner, who brought him up with paternal tenderness.

When about the age of eight years, Jenner was put to school at Wotton-under-Edge, under the Rev. Mr. Clissold. He was next placed under the tuition of the Rev. Dr. Washbourn at Cirencester, where he made a respectable proficiency in the classics, and laid the foundation of some of those friendships which continued throughout life. In the lives of men who attain eminence, we often find, and we are always disposed to look for, indications of that eminence at an early period. We discover, or fancy that we discover, in the child, precocious tastes or talents; and if we have ceased to observe the prodigies of former times, the lambent flame or the harmless serpent playing around the young hero's head, and telling of his future greatness, the admiration of friends and the partiality of biographers produce *their* miracles, more accordant with modern beliefs and appetites. We cannot be surprised if Jenner's taste for natural history began to shew itself early, for natural history is easily and pleasantly pursued by the country school-boy. We shall, therefore hear, without surprise, though not the less with pleasure, that before he was nine years of age, he had made a collection of the nests of the dormouse; and when at Cirencester, he spent the hours devoted by the other boys to play or recreation, in searching for fossils, which abound in the oölitic formation in that neighbourhood.

His scholastic education being finished, he was removed to Sodbury near Bristol, in order to be instructed in the elements of surgery and pharmacy by Mr. Ludlow, an eminent surgeon there. On the expiration of his term with this gentleman, he went to London to prosecute his professional studies under the direction and instruction of the celebrated John Hunter, in whose family he resided for two years, a favourite pupil.

At this time Jenner was twenty-one years of age. John Hunter was forty-two. The latter had been about two years surgeon to St. George's Hospital, had for a much longer period established his menagerie at Brompton for the purpose of prosecuting his philosophical inquiries into the habits and structure of animals, and had already secured much of that public confidence which his vigorous intellect finally acquired for him. Fortunately for Jenner, he became the pupil and the friend of a man, not only remarkable for his philosophical spirit, but possessed of the same tastes and prosecuting the same inquiries as those of Jenner himself. Admiration was heightened by congeniality of pursuits, imitation facilitated by similarity of acquirements, and every thing conspired to render the genius of Hunter of service in determining the career of the future discoverer of vaccination. The influence of the preceptor on the pupil is determined by circumstances which are in some degree accidental—by enthusiasm upon both sides, and particularly by enthusiasm operating on minds of a similar stamp, and with similar tendencies. Whoever is familiar with the writings of Cicero must have seen this finely painted in his references to his preceptor Scævola, the Augur. It is especially the case when the object of pursuit is not a passing one, which loses its charms with the alterations of circumstances, but possesses as much permanence as the objects of human pursuit can have. Such an object is philosophic truth, the polar star of noble minds, which shines when the ignes minores of ambition have set, when wealth, and rank, and personal distinction, have been proved by trial to be vain. There can be little doubt of the fact, and none of the excellence of the sentiments contained in the following passage.

The boldness and independence of Mr. Hunter's character produced deep and permanent effects on the minds of all who witnessed them. Jenner, in particular, felt their power; he saw a master-spirit advancing steadily in that walk of knowledge to which he himself was led by all the predilections of his taste, and all the influence of his early habits. He saw a kind, free, and manly nature devoted to the acquisition of science, and putting away from him entirely the selfish and personal considerations, which are too apt to encumber the researches, and to circumscribe the objects, of less enlightened minds. The heart of Jenner was peculiarly alive to virtues of this kind, and he had moreover an intellect fully capable of appreciating and admiring the other qualities of his master: it was a singular felicity which brought such men together. The pupil not only respected the teacher, but he loved the man; there was in both, a directness and plainness of conduct, an unquenchable desire of knowledge, and a congenial love of truth.

After completing his professional studies in London, he retired from his preceptor's house; but he did not retire from his good-will and affection, nor from his anxious guidance and direction in his scientific pursuits. An uninterrupted epistolary correspondence was kept up between them, till within a short period of Mr. Hunter's death. A very considerable number of his letters have been preserved.

"It was a truly interesting thing," continues Dr. Baron, "to hear Dr. Jenner, in the evening of his days, descanting, with all the fervor of youthful friendship and attachment, on the commanding and engaging peculiarities of Mr. Hunter's mind. He generally called him the 'dear man,' and when he described the honesty and warmth of his heart, and his never-ceasing energy in the pursuit of knowledge, it was impossible not to be animated by the recital, and to perceive that something more than esteem for high intellectual attainments, was required to form that bond of union which, to the last hour of his life, joined the affectionate recollections of the pupil with the memory of the master." 10.

During the time of his residence with Mr. Hunter, in 1771, Captain Cook returned from his first voyage of discovery, The valuable specimens of natural history which had been collected by Sir Joseph Banks, were in a great measure arranged and prepared by Jenner, who was recommended by Mr. Hunter for that purpose. He evinced so much dexterity and knowledge in executing this duty, that he was offered the appointment of Naturalist in the next expedition, which sailed in 1772. But no such offers could tempt young Jenner from his birth-place, endeared to him by the ties of kindred, of habit, and of taste. The religious mind will perceive, and probably with truth, the special interposition of Providence in this. Be that as it may, young Jenner returned to Berkeley, where he rapidly acquired a practice and a reputation which seldom wait upon youth. Yet the labours of the former and seductions of the latter were unable to draw him from his favourite pursuits, and he collected specimens in natural history, and made preparations in comparative anatomy with equal assiduity and success.

" About this period an incident occurred, which might have dissevered his connexion with his native country. He was dining with a large party at Bath, when a question arose whether the temperature was highest in the centre of the flame of a candle, or at some small distance from its apex. Various opinions were delivered, but Jenner, with his usual ingenuity and readiness, soon settled the dispute. He placed the candle before him, and inserting his finger into the middle of the flame, he retained it in this situation for a short time. He then placed it a little above the flame, but was compelled immediately to withdraw it. 'There, gentleman,' he observed, 'the question is settled.'" 12.

Jenner must have been something of the salamander, if this anecdote is to be taken sine grano salis. How the finger is to be got into the middle of the flame, without first passing through the side, is a question not readily answered. And if any body will try to put his finger into the middle of a candle's flame, we fear he will not keep it there long enough to resolve philosophic doubts upon the hottest part of it. We can answer, at all events, for ourselves, and, for curiosity's sake, we have made the experiment. However, Jenner displayed on this occasion, says his biographer, so much talent, that a gentleman of the party offered to procure him an appointment of emolument and distinction in the East Indies. The gentleman thought, probably, that a man who could keep his finger in a flame was very well adapted for a hot climate. But Jenner refused to go.

Dr. Baron dwells with pleasure on Jenner's love of the picturesque, on his manners, his kindheartedness. But the affection of the biographer consecrates details that seem unimportant in the eyes of others, and scarcely carry conviction along with them. The following sketch of Jenner's personal appearance at this period, may perhaps be attended with more general interest.

"His height was rather under the middle size, his person was robust, but active, and well-formed. In his dress he was peculiarly neat, and every thing about him showed the man intent and serious, and well prepared to meet the duties of his calling.

When I first saw him it was on Frampton Green. I was somewhat his junior in years, and had heard so much of Mr. Jenner of Berkeley, that I had no small curiosity to see him. He was dressed in a blue coat and yellow buttons, buckskins, well-polished jockey boots, with handsome silver spurs, and he carried a smart whip with a silver handle. His hair, after the fashion of the times, was done up in a club, and he wore a broad-brimmed hat.

We were introduced on that occasion, and I was delighted and astonished. I was prepared to find an accomplished man, and all the country spoke of him as a skilful surgeon, and a great naturalist; but I did not expect to find him so much at home on other matters. I, who had been spending my time in cultivating my judgment by abstract study, and smit from my boyhood with the love of song, and sought my amusement in the rosy fields of imagination, was not less surprised than gratified to find that the ancient affinity between Apollo and Esculapius was so well maintained in his person." 16.

In Jenner, as in many men of genius, philosophic tastes were blended with convivial qualities; and a warm imagination not inconsistent with the former, lent a zest, and gave an intellectuality to the latter. Had this not been so, Jenner could not have become so general a favourite as undoubtedly he was. His biographer subjoins several specimens of his poetic compositions. We shall introduce one. Though not of the very highest order, it still displays a good deal of spirit, some naiveté, and a fair power of versification.

"ADDRESS TO A ROBIN.

IN ANSWER TO ONE BY CAPTAIN SNELL.

Begone this instant from my door!
Nor plague me with thy canting more.
Hop off! I say, nor in this place
Dare show thy hypocritic face.
Pray do'st thou think, ungrateful fellow,
Because thy voice is somewhat mellow,
Or that thou hither com'st assuming
A kind of modesty in pluming;
Wilt thou allure me, whining beggar?
Or my true notions of thee stagger?

Have I not seen thee, sturdy ruffian,
With impious claw thy father cuffing?*
Seen thee, thou vile impostor, blackguard,
With many a blow thy mother smack hard?
Strip from her back the downy feather,
Spite of inclemency of weather;
Nay, threaten her with instant killing,
If thy full platter she put bill in:
Why then how dar'st thou thus from me
To ask for hospitality?
Disdainful wretch! when smiling Spring
Bids every bird tune up and sing,
Though the sweet orchestra should want ye
To take a part, a soft andante,
The lark, who leads the band, in vain
Solicits thy assisting strain,
For slily thou leav'st all their chanting,
Deep in the woods to go gallanting.
Long have I known thy ready knack 'tis
A thousand wily tricks to practise.
Did'st thou not use deception vile
A bard† to cozen and beguile,
Draw by a kind of *hocus pocus*
His rays poetic to a focus,
Then craftily divert the flame
To blaze upon thy worthless name?
Think'st thou I know not, rogue ungrateful,
Of mischief thou hast got a pateful?
Do qualms of conscience ne'er molest thee?
No retrospective thoughts infest thee?
Hast thou not entered farmer's houses,
Annoying oft their lawful spouses?
Deform'd their butter, peck'd their cheese,
And robb'd them of their market fees?
Though ne'er did they deny thy asking,
(Villain, a hypocritic mask in!)
But ever ready were to pour
Around thy head the crumby show'r.
And pray another thing—but 'sdeath!
Why do I thus consume my breath?"—
Once more I say, Hop off!—hoh! hoh!
'Tis well thou thought'st it time to go:
And this I tell thee, little blade,
If ever on my palisade
Again I catch thee—by the law
Thy grave shall be Grimalkin's maw."

Jenner was an epigrammatist. Here is one of his jeux d'esprit.

On the Death of an old Woman named Heywood.
*Not remarkable for having led an exemplary life.*

Tho' some may exclaim 'Twas strange, or 'twas cruel,
Yet 'tis said to be true; Old Nick wanting fuel,
Gave an order for faggots well-seasoned and good;
So Death took his hatchet, and cut down Hay-wood."

* "Unum arbustum non alit duos Erythacos."
† Captain Snell.

Jenner was very fond of music, and was a member of a catch-club that met at Cam. He could play, and occasionally did so at parties, on the violin and flute. He had a particular dislike to cards. Between the years 1773 and 1783, Jenner was occupied with the duties of his practice, with commissions from John Hunter, with the prosecution of some researches in natural history, and on fossil bones, and with the amusements of his convivial hours. During this period letters appear to have been continually passing between Hunter and himself. His letters to Hunter are lost, those from Hunter to him have been preserved. Dr. Baron presents them to the public, and as they throw light on the pursuits of these eminent men, as well as on the character of Hunter, they are interesting. We cannot attempt to quote all these letters, but we may extract a few passages from them. They are decidedly favourable to Hunter as a letter-writer. The ease, and in some instances the pleasantry of the style contrasts very strongly with the laboured and inflated character of his published works.

In one letter we find the following evidence of the good understanding existing between them :—

"This evening, looking into my book of patients, to scratch out the name of one who had just paid me, and whose name began with an M, I saw a Mr. Mathews, of Berkeley, recommended by you. He did not pay me. I forget whether he was recommended by you as a friend, to serve him, or me: if it was to serve him, I scratch him out of my book. Do you keep an account of the observations of the cuckoo; or must I refer to your letters?—I want a nest with the egg in it; also a nest with a young cuckoo, and also an old cuckoo. I hear you saying, there is no end to your wants.

Ever yours,

JOHN HUNTER."

It appears that Hunter entertained a design of establishing a school of natural history on an extensive scale. He intended teaching natural history, in conjunction with human and comparative anatomy. This implied assistance, and he turned to Jenner. The latter was required to go to London and to pay down a thousand pounds. Jenner declined, and Hunter afterwards abandoned the scheme.

In May 1777, Hunter casually communicated to Jenner the commencement of that illness which sixteen years afterwards carried him off. In the latter end of the year, Hunter was compelled to seek relief in the mild atmosphere of Bath. There Jenner saw him, was shocked, concealed his sentiments from Hunter, but wrote the following letter to Dr. Heberden. The letter deserves notice, because, in it, Jenner first puts forward that theory of the cause of angina pectoris, which has received more credit, than, in all probability, it really merits.

"E. JENNER, TO DR. HEBERDEN.—1778.

Sir,—When you are acquainted with my motives, I presume you will pardon the liberty I take in addressing you. I am prompted to it from a knowledge of the mutual regard that subsists between you and my worthy friend Mr. Hunter. When I had the pleasure of seeing him at Bath last Autumn, I thought he was affected with many symptoms of the Angina Pectoris. The dissections (as far as I have seen) of those who have died of it, throw but little light upon the subject. Though in the course of my practice I have seen many fall victims to this dreadful disease, yet I have only had two opportunities of an examination after death. In the first of these I found no material disease of the heart, except that the coronary artery appeared thickened.

As no notice had been taken of such a circumstance by any body who had written on the subject, I concluded that we must still seek for other causes as productive of the disease: but about three weeks ago, Mr. Paytherus, a surgeon at Ross, in Herefordshire, desired me to examine with him the heart of a person who had died of the Angina Pectoris a few days before. Here we found the same appearance of the coronary arteries as in the former case. But what I had taken to be an ossification of the vessel itself, Mr. P. discovered to be a kind of firm fleshy tube, formed within the vessel, with a considerable quantity of ossific matter dispersed irregularly through it. This tube did not appear to have any vascular connection with the coats of the artery, but seemed to lie merely in simple contact with it.

As the heart, I believe, in every subject that has died of the Angina Pectoris, has been found extremely loaded with fat; and as these vessels lie quite concealed in that substance, is it possible this appearance may have been overlooked? The importance of the coronary arteries, and how much the heart must suffer from their not being able duly to perform their functions, (we cannot be surprised at the painful spasms) is a subject I need not enlarge upon, therefore shall only just remark that it is possible that all the symptoms may arise from this one circumstance.

As I frequently write to Mr. H. I have been some time in hesitation respecting the propriety of communicating the matter to him, and should be exceedingly thankful to you, Sir, for your advice upon the subject. Should it be admitted that this is the cause of the disease, I fear the medical world may seek in vain for a remedy, and I am fearful, (if Mr. H. should admit this to be the cause of the disease) that it may deprive him of the hopes of a recovery." 40.

With admirable delicacy Jenner refrained from publishing the results of his observations on the cause of the angina, lest Mr. Hunter's attention should be drawn to it, and his alarm should be, in consequence, greatly aggravated. As it turned out, Mr. Hunter *had* ossification of the coronary arteries.

About this period Jenner was instrumental in forming a society having for its object the improvement of medical science. Dr. Parry, of Bath, was among the members. The paper of the former on angina pectoris, which forms the ground-work of the book of the latter on the subject, was read at one of the meetings. Jenner also communicated other papers. They fell into the hands of some of the members, and he could never recover them. He regretted the loss of one of them particularly. It contained, says Dr. Baron, observations respecting a disease of the heart, which frequently comes on during attacks of acute rheumatism, and leads to enlargement and disorganization of the part. This formidable disorder had very much escaped the notice of medical men. Jenner's observations were original, and had they been published at the time they were first communicated to the society, his claims to priority could not have been set aside as they have been since that time by other writers.

Jenner was also a member of another society, which assembled at the Ship at Alveston near Bristol. This he called the Convivio-Medical Society, in opposition to the other—the Medico-Convivial. A curious circumstance is mentioned in relation to this Society. Nothing, however, is more probable than that a set of jovial fellows should vote vaccination a bore.

"Dr. Jenner has frequently told me that at the meetings of this society he was accustomed to bring forward the reported prophylactic virtues of cow-pox, and earnestly to recommend his medical friends to prosecute the inquiry. All his efforts were, however, ineffectual; his brethren were acquainted with the rumour, but they looked upon it as one of those vague notions from which no accurate or valuable information could be gathered, especially as most of them had met with cases in which those who were supposed to have had cow-pox, had subsequently been affected with small-pox. These discouragements, as will be seen more at large hereafter, did not suppress the ardour of Jenner's mind. He often recurred to the subject in these meetings; at length it became so distasteful to his companions, that I have many times heard him declare that they threatened to expel him if he continued to harass them with so unprofitable a subject. This society was in existence so late as 1789." 48.

Dr. Baron alludes, in this place, to a claim which has been advanced for Mr. Fewster, of Thornbury, that *he* was the inventor of vaccination. On this head, Dr. Baron makes the following statements.

"That gentleman in his early days was associated with Sutton in the practice of small-pox inoculation, and had frequent opportunities of hearing the popular notion that those who had had cow-pox could not be infected with small-pox. He, as has just been said, was one of the members of the medical society which met at Alveston. The subject was often brought before the meetings, but neither this circumstance, nor his own previous acquaintance with the reports of the country, and his experience, that they were not altogether without foundation, could induce him to prosecute the investigation further, or to countenance Jenner's efforts. On the contrary, he certainly undervalued them, and continued to do so even after the inquiry was published. In a letter to Mr. Rolph, surgeon, in Peckham, dated at Thornbury on the 11th of October 1798, and published in Dr. Pearson's inquiry concerning the history of the cow-pox, he has the following words:—'I think it (*i. e.* the cow-pox in the natural way) is a much more severe disease in general than the inoculated small-pox. I do not see any great advantage from inoculation for the cow-pox: inoculation for the small-pox seems so well understood, that there is very little need of a substitute. It is curious, however, and may lead to improvements.'" 49.

This latter passage is conclusive. The passive resistance of Mr. Fewster would never have spread vaccination over the world. There cannot be much doubt upon that head.

Not long after this, Jenner appears to have met with one of those tender disappointments which would seem to be frequently the lot of men of genius. Dr. Baron offers no more explicit account of it than is contained in a jocose letter of Mr. Hunter on the occasion: we are afraid that Hunter will find little favour with the ladies. He seems to have made small-beer of a love affair. Mrs. Hunter, herself, was a very namby-pamby sort of person.

"MR. HUNTER TO E. JENNER.

Dear Jenner,—I own I was at a loss to account for your silence, and I was sorry at the cause. I can easily conceive how you must feel, for you have two passions to cope with, viz., that of being disappointed in love, and that of being defeated, but both will wear out, perhaps the first soonest. I own I was glad, when I heard that you was married to a woman of fortune; but let her go, never mind her. I shall employ you with hedge-hogs, for I do not know how far I may trust mine. I want you to get a hedge-hog in the beginning of winter and weigh him; put him in your garden, and let him have some leaves, hay, or straw, to cover himself with, which he will do: then weigh him in the spring, and see what he has lost. Secondly, I want you to kill one at the beginning of winter to see how fat he is, and another in the spring, to see what he has lost of his fat. Thirdly, when the weather is very cold, and about the month of January,

I could wish you would make a hole in one of their bellies, and put the thermometer down into the pelvis, and see the height of the mercury; then turn it up towards the diaphragm and observe the heat there. So much at present for hedge-hogs. I beg pardon, examine the stomach and intestines. If Hewson's things go cheap, I will purchase some that I think proper for you; those you mention I am afraid will be every body's money, and go dear.

Ever yours,

*London, Sept. 25th,* 1778. JOHN HUNTER."

Certainly hedgehogs are a novel recipe for disappointed love. They are such, perhaps, as a naturalist might hit upon, but we doubt whether they would be generally efficacious. They do not seem to have succeeded particularly well with Jenner, for, five years afterwards, he writes:—"I am jaded almost to death, my dear Gardner, by constant fatigue: that of the body I must endure; but how long I shall be able to bear that of the mind, I know not. Still the same dead weight hangs upon my heart. Would to God it would drag it from its unhappy mansion! then with what pleasure could I see an end of this silly dream of life."

This sentiment is pretty well for a member of the Medico-Convivial and the Convivio-Medical Societies. Not unfrequently however, persons of a social turn of mind feel disappointments of this description keenly. They are social, because the kindlier feelings of life are necessary to their comfort or their happiness, and the closest and kindliest tie of all has peculiar charms for them. The philosopher, absorbed in the contemplation of Nature, looks on the charities and the affections of humanity as an integral part of her scheme, and reasons statistically on the loss of a wife or a child.

Jenner was a collector of fossil remains. He co-operated with Mr. Hunter in his experiments on the hybernation of animals, on the cuckoo, and on various other subjects. Amongst others, he instituted a new formula for the preparation of emetic-tartar, on which Mr. Hunter wrote the following letter.

"Dear Jenner—I am puffing off your tartar, as the tartar of all tartars, and have given it to several physicians to make trial, but have had no account yet of the success. Had you not better let a bookseller have it to sell, as Glass of Oxford did his magnesia? Let it be called Jenner's Tartar Emetic, or any body's else you please. If that mode would do, I will speak to some, viz. Newbery, &c. You are very sly, although you think I cannot see it: you very modestly ask for a thermometer; I will send one, but take care that those d——d clumsy fingers do not break it also."

J. HUNTER."

In the commencement of 1786, Jenner incurred great hazard from exposure to cold. He describes the occurrence very graphically himself.

"January 3d, 1786.—I was under the necessity of going from hence (Berkeley) to Kingscote. The air felt more intensely cold than I ever remember to have experienced it. The ground was deeply covered with snow, and it blew quite a hurricane, accompanied with continual snow. Being well clothed, I did not find the cold make much impression upon me till I ascended the hills, and then I began to feel myself benumbed. There was no possibility of keeping the snow from driving under my hat, so that half my face and my neck was, for a long time, wrapt in ice. There was no retreating, and I had still two miles to go, the greatest part of the way over the highest downs in the country. As the sense of external cold increased, the heat about the stomach seemed to increase.

I had the same sensation as if I had drank a considerable quantity of wine or brandy; and my spirits rose in proportion to this sensation.

I felt, as if it were, like one intoxicated, and could not forbear singing, &c. My hands at last grew extremely painful, and this distressed my spirits in some degree. When I came to the house I was unable to dismount without assistance. I was almost senseless; but I had just recollection and power enough left to prevent the servants from bringing me to a fire. I was carried to the stable first, and from thence was gradually introduced to a warmer atmosphere. I could bear no greater heat than that of the stable for some time. Rubbing my hands in snow took off the pain very quickly. The parts which had been most benumbed, felt for some time afterwards as if they had been slightly burnt. My horse lost part of the cuticle and hair at the upper part of the neck, and also from his ears. I had not the least inclination to take wine, or any kind of refreshment.

One man perished a few miles from Kingscote, at the same time, and from the same cause." 73.

Jenner prosecuted some experiments on animal manure, and instituted very careful observations on the habits of the cuckoo. These were published in the Transactions of the Royal Society, and are creditable to the accuracy and patience of the observer. But in 1788, he had an opportunity of studying the habits of a higher animal than a cuckoo—a wife. He married a Miss Catherine Ringcote, a lady to whom he had been long attached, and a member of an ancient and respectable family. On the 24th of January, 1789, his eldest son Edward was born. He appears to have been very happy with his bride, for he writes to his friend Gardner—" My place of residence, though unfinished, is extremely comfortable; and I can with truth assure you the last year of my life, dating it from the month of March, has been the happiest beyond all comparison I ever experienced: and I will take upon me to aver (nay I would swear it) that if you could be lucky enough to connect yourself with a woman of such a disposition as kind fortune has, at last, given to me, you will find a vast addition to your stock of happiness." We are almost tempted to quote some other passages illustrative of Jenner's kindly disposition. But that would, after all, be needless. Every page of his history speaks the same language upon that point. We shall introduce a passage or two illustrative of his good sense in the conduct of life—as important a requisite for success and happiness as any. Writing to a friend of a child, he observes:—" I hear by the captain, you and Mrs. Clinch keep him under no kind of restraint, but indulge him with a full gratification of all his wants and wishes. Let me entreat you both to take care what you are about:— remember the path of life is full of *thorns*, and if you keep him upon *velvet* till the day arrives when he must begin to feel their points, think how much more poignant must be his feelings. On this subject one might enlarge, but what I have said I trust will be sufficient to awaken every proper sentiment. I shall only just add that severity to children I utterly abhor, and my observation leads me to consider it as being more injurious to them than the contrary extreme; but either one or the other is so hurtful to the mind that we feel its baneful effects to the very latest period of our lives."

Jenner's experience coincided with that of most of us when he remarked—

" Henry is much as you left him, the same simple, inoffensive lad, and indeed, I think as boyish almost as ever; and though his mind is stored with ideas that do him the greatest credit, yet his general appearance and manner is so very *fifteenish*, that a poor mortal on the bed of sickness will hardly look up to him with that eye of confidence and hope that might safely be placed in him. For it is by appearances, my dear friend, not from a real knowledge of things, that the world (at least the major part of them) form a judgment. A look of significance, a peculiar habit, and a very scanty acquaintance with the human machine, will make a man pass current for a great physician. This you and I know to be an unfortunate fact; while, without these auxiliaries, a man with the knowledge of the *hall* and *college* concentrated will be looked upon as a mere pretender." 92.

In a newspaper criticism on Mr. Pettigrew's Lives of eminent Physicians and Surgeons, the sagacious editor observed how much more scientific the ancient physicians looked than the modern. Our predecessors wore large wigs, carried ponderous canes, shook their heads dismally, and indulged in the dead languages. Of course they must have possessed more medical science than ourselves.

Mr. Hunter died on the 12th of August, 1793. He happened to *have* that ossification of the coronary arteries, on which Jenner had not ventured to touch, with his valued preceptor and friend. The fatigues of general practice having become irksome to Jenner he resolved to abandon one branch of it, and to confine himself to medicine. With that view he obtained in 1792 a degree of Doctor of Physic from St. Andrew's. Towards the conclusion of 1794, he was attacked with typhus fever, a complaint to which he appears to have been prone, and nearly sank under it.

In the Spring of 1796, he performed his first inoculation of the *variolæ vaccinæ*, and in the course of the year 1797, he had nearly arranged every thing for the publication of his Inquiry. The history of vaccination becomes from this time the history of Jenner. We shall only go into the former far enough, to exhibit the progress of most great discoveries, and to show the time and labour consumed in rendering the world sensible of the acquisition of a valuable boon. Such a history is always useful. It stimulates the dull, animates the quick, and encourages young minds, which are too apt to be disheartened at the depth of the well in which truth lies hid, and the labour of bringing it up. It is a pleasant task to do justice to the dead, and at the same time to encourage the living, to honour departed genius, and to nurse the coming. And a sketch of the history of vaccination will do this.

The attention of Jenner was drawn to the nature of cow-pox while he was yet young. The student's mind contained the germ of the discovery of future years, and the seed of immortality was early and simply sown.

"He was pursuing his professional education in the house of his master at Sodbury: a young country-woman came to seek advice; the subject of small-pox was mentioned in her presence; she immediately observed, 'I cannot take that disease, for I have had cow-pox.' This incident rivetted the attention of Jenner. It was the first time that the popular notion, which was not at all uncommon in the district, had been brought home to him with force and influence. Most happily the impression which was then made was never effaced. Young as he was, and insufficiently acquainted with any of the laws of physiology or pathology, he dwelt with deep interest on the communication which had been casually made known to him by a peasant, and partly foresaw the vast consequences which were involved in so remarkable a phenomenon. He was the more stimulated to meditations of this sort by frequent opportunities of witnessing the ravages of small-pox; and by retaining the most vivid and painful recollections of the severe discipline which he himself had not long before passed through, preparatory to his inoculation for that disease. 'There was,' to use his own words, 'bleeding till the blood was thin; purging till the body was wasted to a skeleton; and starving on vegetable diet to keep it so.' The possibility of averting such evils could not arise in a mind like Jenner's without possessing it fully; and he resolved to let no opportunity escape of acquiring knowledge on so important a subject." 123.

He mentioned it to John Hunter. The latter was not a man to throw cold water upon scientific discoveries. His attention was too much occupied with inquiries of his own, to permit him to do more than to listen to Jenner. But he made known Jenner's opinions, as well as the Gloucestershire traditions, to his pupils in his lectures, and to his friends in conversation. And he encouraged Jenner to put the matter to the test of experiment. "Don't *think*," said Hunter, "but *try*; be patient, be accurate." A golden sentence, comprising all the spirit of genuine philosophy. But others did throw cold water on the embryo investigation, and more than the members of the Medico-convivial Society voted it a bore. In 1780, the following circumstance occurred.

"He was riding with Gardner, on the road between Gloucester and Bristol, near Newport, when the conversation passed of which I have made mention. He went over the natural history of cow-pox; stated his opinion as to the origin of this affection from the heel of the horse; specified the different sorts of disease which attacked the milkers when they handled infected cows; dwelt upon that variety which afforded protection against small-pox; and with deep and anxious emotion mentioned his hope of being able to propagate that variety from one human being to another, till he had disseminated the practice all over the globe, to the total extinction of small-pox. The conversation was concluded by Jenner in words to the following effect:—'Gardner, I have entrusted a most important matter to you, which I firmly believe will prove of essential benefit to the human race. I know you, and should not wish what I have stated to be brought into conversation; for should any thing untoward turn up in my experiments I should be made, particularly by my medical brethren, the subject of ridicule—for I am the mark they all shoot at." 129.

How striking a lesson does this short speech of Jenner's read—how chastening to human intellect. The mention of the greatest benefit that has ever been conferred upon humanity was generally pronounced a nuisance—the whole subject was a joke—the author of it a butt. How chary should we be in the use of ridicule, as a means of determining philosophic truth—how suspicious of our powers of resolving at once the real value of new facts or strange opinions.

We shall not pursue the investigations, the doubts, the confirmations of Jenner. They led him at last to the belief, all but assured, of the powers of the disease when communicated from the cow, to destroy the liability to small-pox. But the question that arose was the communicability of the protective malady from one *person* to another. An important question. An opportunity occurred on the 14th of May, 1796, of instituting this experiment. Matter was taken from the hand of Sarah Nelmes who had been infected by her master's cows, and inserted by two superficial incisions into the arms of James Phipps, a healthy boy of about eight years old. He went through the disease apparently in a regular and satisfactory manner. But this was not all. It was still to be ascertained whether this complaint, communicated by art from one individual to another, was really protective against small-pox. This was to be determined by positive experiments. How much hung upon the issue, how anxious the mind of the experimenter must have been, may be guessed, but cannot be told.

On the first of July, Jenner inoculated Phipps with variolous matter. No disease followed. He communicated the event to his friend Gardner in the following letter.

"Dear Gardner,—As I promised to let you know how I proceeded in my inquiry into the nature of that singular disease the cow-pox, and being fully satisfied how much you feel interested in its success, you will be gratified in hearing that I have at length accomplished what I have been so long waiting for, the passing of the vaccine virus from one human being to another by the ordinary mode of inoculation.

A boy of the name of Phipps was inoculated in the arm from a pustule on the hand of a young woman who was infected by her master's cows. Having never seen the disease but in its casual way before; that is, when communicated from the cow to the hand of the milker, I was astonished at the close resemblance of the pustules, in some of their stages, to the variolous pustules. But now listen to the most delightful part of my story. The boy has since been inoculated for the small-pox which, as I ventured to predict, produced no effect. I shall now pursue my experiments with redoubled ardour.

Believe me yours, very sincerely,

*Berkeley, July* 19, 1796. EDWARD JENNER."

It requires little knowledge of human nature to imagine what Jenner's feelings must have been in this stage of his investigation. Believing himself the possessor of a secret of the first consequence to the human race—trembling at every fresh experiment, lest it should damp his hopes—anxious to communicate what he knew to the world, yet fearing to compromise himself, his cause, and the truth, by precipitancy, his bosom must often have been the seat of the most conflicting feelings. He says himself:—"While the vaccine discovery was progressive the joy I felt at the prospect before me of being the instrument destined to take away from the world one of its greatest calamities, blended with the fond hope of enjoying independence and domestic peace and happiness, was often so excessive that, in pursuing my favourite subject among the meadows, I have sometimes found myself in a kind of reverie. It is pleasant to me to recollect that these reflections always ended in devout acknowledgments to that Being from whom this and all other mercies flow."

The evidence was sufficiently collected and consolidated to be laid before the public. It was given in the form of a quarto, about the end of June, 1798. It was dedicated to Dr. Parry, of Bath, and very little exceeded seventy pages. This form of publication was judged more adviseable than a Paper in the Transactions of the Royal Society.

Just prior to this, Jenner repaired to London, for the purpose of exhibiting the cow-pox. He remained in town for nearly three months, without being able to procure one person on whom he could exhibit the vaccine disease. He often stated that his patience had been exhausted on that occasion, and that he had actually quitted the capital without having accomplished the object of his journey.

But some of the virus was consigned to Mr. Cline, who inserted it by two punctures into the hip of a patient. This fact, and a letter of Mr. Cline's upon the subject, deserve to be put upon record, for they shew the sagacity and philosophical spirit of that surgeon in a favourable light.

"COPY OF MR. CLINE'S LETTER.

*Lincoln's-Inn Fields, 2d Aug.* 1798.

'The cow-pox experiment has succeeded admirably. The child sickened on the seventh day; and the fever, which was moderate, subsided on the eleventh day. The inflammation extended to about four inches diameter, and then gradually subsided without having been attended with pain, or other inconvenience. The ulcer was not large enough to contain a pea, therefore, I have not converted into an issue as I intended.* I have since inoculated him with small-pox matter in three places, which were slightly inflamed on the third day, and then subsided.

Dr. Lister, who was formerly physician to the Small-pox Hospital, attended the child with me, and he is convinced that it is not possible to give him the small-pox.

I think the substituting of cow-pox poison for the small-pox promises to be one of the greatest improvements that has ever been made in medicine: for it is not only so safe in itself, but also does not endanger others by contagion, in which way the small-pox has done infinite mischief. The more I think on the subject the more I am impressed with its importance.

With great esteem, I am, dear Sir,

Your faithful servant,

HENRY CLINE.'"

Cline advised Jenner to repair to London immediately, and to take a house in Grosvenor-Square, asssuring him of an income of £10,000 a year by practice. The late Sir W. Farquhar coincided with Cline in the advice and the anticipation. But Jenner was proof against the temptation. He was not anxious for gold, he shunned the angry competition of Metropolitan practice, and his ambition was of a nobler order, than that which wealth would satisfy. "My fortune," said he, "with what flows in from my profession, is sufficient to gratify my wishes; indeed so limited is my ambition and that of my nearest connexions, that were I precluded from future practice I should be enabled to obtain all I want. And as for fame what is it? a gilded butt, for ever pierced with the arrows of malignancy."

Jenner received many letters from men of eminence, among others from Dr. Percival, Dr. Haygarth, and Dr. Hicks, congratulating him on his discovery, and urging him to render it as perfect as investigation could make it. All men were not opposed to vaccination because it was novel, but those of superior minds were generally disposed to submit it to the test of inquiry, and to extend to it the advantage of patronage.

We pass by four chapters occupied with discussions on the history of variola, variolæ vaccinæ, and varioloid diseases, and we take up the thread of Jenner's life after the publication of his "Inquiry."

The celebrated Dr. Ingenhousz going on a visit to the Marquess of Lansdown at his seat in Wiltshire, made it his business to inquire among the dairies, where cow-pox sometimes prevailed, respecting its reported virtues

* "This boy was brought to town on account of some disease in the joint of the hip. Mr. C. therefore inoculated near the part, with the view of exciting inflammation, and subsequently of forming an issue. E. J."

as a preventive of small-pox; and he got, as might have been expected, such answers as Dr. Jenner had obtained at the commencement of his investigation. Some individuals, who had had what was called cow-pox, were subsequently affected with small-pox. It cannot be a matter of surprise that Ingenhousz should be influenced by the facts he heard, rather than by what must necessarily have appeared the hypothetical explanations of Jenner. It is not fair to judge by the event, and there were many circumstances connected with the origin of vaccination calculated to inspire doubt. Jenner believed that vaccination was a certain preventive against small-pox. Facts appeared on inquiry to contradict that confident assertion. Do they confirm it even *now*? We are afraid not. Jenner was much annoyed and more excited. The commencement and conclusion of a letter, shew his condition of mind.

"Dear Gardner,—I fully depend upon meeting you at Eastington to-morrow to sit in council on several subjects of high import. My friends must not desert me now. Brickbats and hostile weapons of every sort are flying thick around me; but with a very little aid, a few friendly opiates seasonably administered, they will do me no injury."

"My experiments move on—but I have all to do single-handed. Not the least assistance from a quarter where I had the most right to expect it!!

Bodily labor I disregard, but pressures of the mind grow too heavy for me. Added to all my other cares, I am touched hard with the reigning epidemic—impecuniosity. Any supplies from the paper-maker?" 297.

A new source of annoyance however developed itself, as unexpected, as dangerous. It was not the opposition of enemies, but the interested assistance of friends. Cow-pox broke out among some cows in Gray's Inn Lane. Many London Physicians ran to observe, to experiment, to fail. To fail, because they operated with vaccine matter at the Small-Pox Hospital, in such a way that the two poisons got mixed up together, and a sort of varioloid vaccine lymph was used in London and sent abroad. The result, of course, was confusion. But this was not all. Dr. Pearson became extremely busy in vaccinating, and preaching vaccination, and evinced every inclination to step quietly into Jenner's shoes. The following is an extract from a letter of George Jenner to the Doctor.

"I shall only state a few facts I have got possession of since I wrote to you last. Dr. Pearson is going to send circular letters to the medical gentlemen to let them know that he will supply them with cow-pox matter upon their application to him, by which means *he* will be the chief person known in the business, and consequently deprive you of that merit, or at least a great share of it, which is so justly your due. Doctor P. gave a public lecture on the cow-pox on Saturday last. Farmer Tanner was there. Doctor Pearson adopted your opinions, except with regard to the probability of the diseases originating in horses' heels. He spoke of some unsatisfactory experiments having been made by inoculating from the greasy heels; but when we consider how difficult it was to communicate the disease from one cow to another by inoculation, we are not to wonder at the still greater difficulty in communicating it from the horse to the cow. The farmer says Dr. Pearson was wrong in some part of his lecture, which he *took the liberty to tell him.*" 319.

Jenner may well be supposed to have been annoyed at this. He was so. Other attacks too, and other circumstances, conspired to augment his anxiety and chagrin. What he felt he expressed in a letter to Mr. Edward Gardner, a portion of which we shall quote.

"Dear Gardner,—There never was a period in my existence when my situation called so loudly for the assistance of my literary friends as the present. Though my bark will, with flying colours, reach the shore at last, yet it is now in a storm.

I am beset on all sides with snarling fellows, and so ignorant withal that they know no more of the disease they write about than the animals which generate it. The last philippic that has appeared comes from Bristol, and is communicated by Dr. Sims of London. Sims gives comments on it in harsh and unjustifiable language. It is impossible for me, single-handed, to combat all my adversaries.

Standing, as I do, before so awful a tribunal, my friends will volunteer their counsel and IMMEDIATELY appear in court.

My intended pamphlet has only been looked over in a cursory way. Every sentence must be again revised and weighed in the nicest balance that human intellect can invent." 322.

On the 21st of March, 1799, Jenner arrived in London, and took up his residence in Norfolk-street. He had an interview with Dr. Woodville, and was tolerably well occupied in correcting the blunders that had arisen from the mixing up of the small-pox and vaccine poisons in town. This latter circumstance gave birth to the idea that vaccination *might* give rise, and often did, to a smart eruptive disease. Nay Dr. Woodville concluded that it had proved fatal once in 500 cases, a mortality greater than that from inoculated small-pox, which had appeared to be one in 600. We need scarcely say that logical as this reasoning seemed, it was fallacious, because the data themselves had been erroneous.

But notwithstanding, says Dr. Baron, the prejudices which were excited against cow-pox by the report of its being an eruptive disease, it continued to gain ground. Many distinguished individuals, in different parts of the kingdom, eagerly availed themselves of the promised benefit of the practice, and exerted themselves to diffuse it as widely as possible. It is a gratifying thing to be able to remark that the ladies of England were conspicuous in this work. Lady Peyton, sister of Lord Rous, must be ranked among the first who, by personal efforts, stimulated the professional gentlemen in her neighbourhood to adopt vaccination; and she herself subsequently became one of the most energetic and successful of vaccinators.

Jenner returned to Berkeley, and what between vaccinating, forwarding vaccination, and correcting the bad consequences of the mixture of the London vaccine lymph with small-pox matter, he had business enough upon his hands. We cannot enter into particulars, but it is remarkable how great a risk vaccination ran, how narrowly it escaped being damaged by the injudicious interference of its friends. However, the Duke of Clarence's children were vaccinated, the King sent "a very civil message" to the vaccinators, and the Princess Louisa, of Prussia, applied to Jenner for lymph.

It must not be supposed that no active opposition was encountered by Jenner. Dr. Moseley, physician to Chelsea Hospital, thought fit, in a treatise on sugar, to bring forward an irrelevant attack on cow-pox, and then gave his professional brethren a specimen of that elegant and classical phraseology which so peculiarly distinguished his subsequent lucubrations. His station, much more than the force of his argument, gave weight to his observations.

He wrote in great ignorance of the subject; and with all the bitterness and prejudice that generally attend on ignorance.

Mr. Ring very successfully exposed and refuted his remarks. The doctor had seen in distant prospect an awful aggravation of human ills, from an admixture of bestial humours, which the cow mania, as he elegantly termed it, threatened to inflict upon our race. He even predicted an alteration in "the human form divine," and that another brood of minotaurs would overspread the land—

"Semibovemque virum, semivirumque bovem."

Cases were, we believe, published, in which vaccinated persons bellowed like cows, became covered with hair, and even exhibited horns and a tail. It would be highly amusing, but very unprofitable, to collect these *facts*, and to repeat the arguments of Moseley and his coadjutors. But the prejudiced knaves who coined, and fools who credited these bug-bears, are too ridiculous for further notice.

We have seen Dr. Pearson kindly patronising Dr. Jenner. A continuation of this patronage was now extended to him, and very flattering it was. The Doctor, in his anxiety to immortalize his friend, set on foot a vaccine board, of which the chief place was allotted to himself,—the inferior departments being also filled up in conformity with his wishes. The board, thus constructed, received a degree of countenance worthy of a metropolitan charity: His Royal Highness the Duke of York having permitted himself to be named as patron. Lord Egremont also consented to hold an office in it. Matters being thus arranged, it became the business of Dr. Pearson to announce the matter to Jenner. Our readers may conceive the delight with which the former must have seized the opportunity of honouring the discoverer of vaccination—the elevated post he would assign to him. Whatever their expectations may be, the fact we venture to say will outrun them. Here is the letter announcing the Institution.

"Dr. Pearson to Dr. Jenner.

*London, Dec. 10th, 1799.*

My dear Sir,—I wish ever to be governed in life by the rule of doing justice; and, if I can, acting liberally to my fellow labourers. I trust I have acted consistently to you; and, if I have differed in opinion on some points, it was because new lights broke in upon me; but I trust in such instances I, too, acted consistently and was more anxious to bestow commendation than to be studious to point out faults. Agreeably to my principle I now address you to say that we have made some progress in the institution of a charity for inoculating the vaccine pock. I do not know that I can confer any honour on you by proposing you (if I am able) to the directors of our establishment, nor do I well know what to propose to you. It occurs to me that it might not be disagreeable to you to be an extra corresponding physician, and I can see no objection to this proposal at our meeting. The medical establishment consists of two physicians of the college, two consulting physicians, two surgeons, and three visiting apothecaries. We have got very high patronage, but the institution is not yet completely organized, nor will be so for some time. Rush, Keate, and his nephew, Gunning, Brand, Devaynes, belong to the medical departments. Exactly what Woodville will or can be, on account of his connexion with the Small-pox Hospital, I cannot tell: but he authorizes me to say in a letter from him, 'that he wishes to give his assistance and promote the undertaking.'

No expense is to be attached to your situation except a guinea a year as a subscriber, and indeed I think you ought to be exempt from that, as you cannot send any patients: but you may depute some proxy in town. I confess I was surprised that you neither called nor sent to me for the last *two* months you were in town. However, if it was because you were so much occupied, I certainly excuse you. I hope you will excuse haste in this letter, but it will serve to assure you that I remain, with great consideration,

Your's truly, G. PEARSON.

Compliments of Mrs. P. and myself to Mrs. Jenner."

The generosity of the man is equalled by the liberality of the patron. Could any thing be more just, could any thing be more honourable, than making Jenner an extra-corresponding physician, and letting him off one whole guinea a year as subscriber. Think of that! made a guinea governor for nothing.

The odd thing is that Jenner did not feel the compliment. He could not perceive that he was amply recompensed for years of anxiety, and for all the toils of investigation and discovery, by the title and office of an extra corresponding physician to another man's institution. Certain it is that he was not sensible of the honour done to him, and he declined it forthwith.

Jenner thought it was time to return to London, which he did on the 28th of January, 1800. His main object was to deliberate with Lord Egremont and other friends respecting the establishment of a vaccine institution. Jenner was introduced to the Duke of Clarence. This was followed by an interview with the Duke of York. The consequence was, that Lord Egremont and the Duke withdrew from Pearson's institution. Dr. Baron remarks that Dr. Jenner's firmness and prudence in this affair gave unqualified satisfaction to his friends; and supported as he was by the handsome and efficient interposition of Lord Egremont, he was enabled to defeat the ambitious designs of those who sought for high patronage in proceedings of a very questionable nature.

"It certainly was the feeling of all those elevated personages who wished to assist in forming a vaccine institution that none could be established which did not assign to the author of the discovery that situation of dignity and influence which was due to his merit, and which would enable him to direct the practice with vigour and effect. This feeling was strongly evinced when the Royal Jennerian Society was formed; and it was also very characteristically expressed at this time by his friend the late munificent Mr. Angerstein. He said, '*that he would not mind a subscription of one hundred or two hundred pounds in an institution organized by the man who was best competent to set about it, but that he would have nothing to do with one grafted on the present blunders.*'" 372.

On the 7th of March, Jenner went to St. James's with Lord Berkeley, and presented his Treatises on Cow-pock to the King. Jenner writes in allusion to this intended presentation—"Pray acquaint Lord Berkeley I shall be ready to accept his kind offer of accompanying me to St. James's any day he may appoint in the course of the week after this. The work will then be finished, and clad in crimson. What will you give for a sight of me all in velvet, girt with a sword too? What a queer creature is a human being!" Towards the end of the month, he had a private interview with the Prince of Wales, and soon afterwards he went to the Queen's drawing-room. So Jenner went the round of the Royal Family, and must have begun to think himself—

"A marvellous proper man."

Writing about this time, he says, "I have not made half my calls yet in town, although I fag from eleven till four." He goes on to observe—"Pray write without delay to Tierney, and tell him how rapidly the cow-pox is marching over the metropolis, and, indeed, through the whole island. The death of the three children under inoculation with the small-pox will probably give that practice the Brutus-stab here, and sink for ever the tyrant small-pox. Would Tierney like to have a little virus, that the cow-pox inoculation may be set going under his own eye at Edinburgh? I should be happy to furnish him. Let him know that my new edition mentioning his name, with the appendix, is published. A very little attention would place the practice in its proper light in Edinburgh, a thing devoutly to be wished." Tierney is the present Sir Matthew Tierney.

In a letter from Mr. Gooch to Dr. Jenner, an instance is mentioned of the prejudice that prevailed against inoculation in some parts. "The first people," he says, "we inoculated in Hadleigh were absolutely pelted, and drove into their houses, if they appeared out; we have now persuaded our apothecary to inoculate the whole town (7 or 800 persons) and our hundred-house is now under inoculation (about 350 persons.)"

But, after all, vaccination met with less opposition than could have been anticipated, considering the strange nature of the fact, its want of analogy with what we know of other morbid poisons, and the numerous mistakes and mishaps that waited on it at the outset. If, even now, we are inclined to doubt whether Jenner was not too sanguine and too peremptory, how much more sceptical must have been the reasoners of that day. We confess that we are surprised both at the boldness and the liberality of such a testimonial as the one we shall adduce signed by such men as Dr. Wall, Chemical Professor of the University of Oxford; Dr. Williams, Regius Professor of Botany; Sir Christopher Pegge, Reader in Anatomy; and Mr. Grosvenor, Surgeon to the Radcliffe Infirmary. The document runs thus:—"We, whose names are undersigned, are fully satisfied, upon the conviction of our own observation, that the cow-pox is not only an infinitely milder disease than the small-pox but has the advantage of not being contagious, and is an effectual remedy against the small-pox."

We shall not stop to trace the progress of vaccination in this country or abroad. In our opinion, it was astonishingly rapid. We prefer accompanying Jenner, who returned to Cheltenham in July, 1800, but in November brought up his family to London. Dr. Baron informs us that his own personal endeavours were, as heretofore, laborious. He offered gratuitous inoculation to all the poor who thought fit to apply at stated periods. His benevolent invitations were, in the main, very generally accepted, parents bringing their children in great numbers both from the town and the adjoining parishes.

Dr. Baron adds an amusing though a not unparalleled incident.

"Notwithstanding his repeated notices of gratuitous aid one parish had hitherto obstinately held back. This year, however, he found the people bringing their children in great numbers. Of course he wished to know by what means they had become converts to the new inoculation. He found that arguments of a very authoritative nature had brought about the change. The small-pox, in the course of the preceding year, had been introduced into the parish, and proved extremely fatal; but it was not this circumstance, nor yet the security of those who had been vaccinated in the adjoining parishes, that brought cow-pox inoculation into favour. The cost of coffins for those who were cut off by small-pox proved burdensome to the parish; the churchwardens, therefore, moved by this argument effectually exerted their authority, and compelled the people to avail themselves of Dr. Jenner's kind offer." 434.

He had again in London a typhoid attack, which he always dreaded. But he was soon able to resume his active duties. His vaccinations among the higher ranks were very numerous; and many of the nobility were desirous of having from his own mouth information on the great question which then engrossed so much of the public attention. A party of this kind assembled at the house of Earl Spencer on the twentieth of December. Besides his Lordship and the Countess there were present Lord Lucan, Lord Camden, Lord Macartney, Mr. Grenville, &c. &c. Jenner, whether in conversation or in writing, never failed to treat this or any other subject, with great eloquence and perspicuity.

Jenner conceived the idea that by vaccinating the dog, that animal might be rendered proof against the "distemper." Accordingly we find him vaccinating twenty of the king's stag-hounds. It would appear that this was an idle notion.

Dr. Jenner has often been blamed, says Dr. Baron, for encouraging unprofessional persons to practise vaccination. Every individual in this situation, with whom he had to do, actually studied the subject, and implicitly followed his directions. Not so many of his professional brethren. They would not condescend to be instructed, and they openly disregarded his rules. Of these one of the most important was violated by the first public body that was formed in London for the purpose of vaccination. They actually printed and distributed a paper stating that the virus may safely be taken from the pustule, so late as the thirteenth day!!!

It certainly would have been desirable to have confined the practice from the beginning to the hands of medical men. It is, nevertheless, true, that, from the causes just mentioned, *they* fell into great error, and did thereby much more endanger the character of vaccination than those who, from their general ignorance of medical subjects, might have been considered as more liable to go astray.

On the 22nd of April, Jenner was admitted a Fellow of the Lyceum Medicum Londinense, a society founded by Hunter and Dr. Fordyce. But now and then some sturdy opponent of vaccination started up. Ehrmann, of Frankfort in an especial manner rendered himself conspicuous by the violence and extravagance of his opposition to the new practice. A learned divine of the church of England (Massey) who preached a sermon against small-pox inoculation, in London, 1722, announced it as no new art, inasmuch as Job, he asserted, had been inoculated by the devil. Ehrmann took rather a bolder flight, and attempted to prove from quotations of the prophetical parts of Scripture, and the writings of the fathers of the church, that the Vaccine was nothing less than Antichrist. The harsh and unsparing tone of his writings obtained for him the appellation of the *Marat* of the Vaccine.

As a set off against Ehrmann, we may take the Empress of All the Russias. On the 10th of August, 1802, she sent Jenner a letter, signed with her own hand, together with a valuable diamond ring. Her letter is really a fair sample of royal good sense and good feeling.

Monsieur Jenner!

D'usage de la Vaccine en Angleterre ayant eu les succès les plus avantageux et les mieux attestés, je me suis empressée d'imiter cet exemple, en l'introduisant dans les establissemens pieux, qui sont sous ma direction. Mes soins remplissant parfaitement mon attente, je me plais à en rapporter le succès, et à en temoigner ma reconnaissance à celui, qui a rendue à l'humanité ce service signale. Ce motif m'engage, Monsieur, à vous offrir la bague ci-jointe comme une témoinage des sentimens d'estime et de bienveillance, avec les quels je suis votre affectionée,

*Powlosk ce* 10 *Août,* 1802. Marie.

In America, the most enlightened of her citizens supported, some of his own profession opposed Jenner. It cannot excite astonishment that John Adams should have countenanced him. The friend of his species, his heart was disposed and his head was qualified to hail and appreciate whatever was beneficial to it. Nor did Jefferson disdain to turn vaccinator. He and his sons-in-law vaccinated in their own families and in those of their neighbours, nearly two hundred persons.

We have arrived at an important epoch in Jenner's life. By dint of untiring efforts, vaccination had become widely known, and was almost as widely practised—it had taken root in all quarters of the globe, and time only was necessary for its culture and maturity—the name of Jenner was pronounced and revered in every civilized and almost every barbarous tongue—his country was honored in himself—and we naturally inquire what was the condition of the man who had done so much for humanity.

He had materially impaired his professional income as well as his private fortune. His frequent journeys to London, indispensable for the success of the cause of vaccination, had been a source of great expense, the extensive correspondence in which he was engaged was also burdensome, his time was occupied, his attention absorbed in the development of his discovery, and, finally, its unreserved diffusion, prevented his receiving any material personal advantage from it. These were not the cheap sacrifices of a wealthy man, the philanthropy which while it gratified the heart did not pinch the body. Jenner had a family and many relatives dependent on him, and while he was studying to preserve the lives of others it was requisite that he should live himself.

Under these circumstances, says his biographer, it was thought that the magnitude of his discovery and the very disinterested manner in which he was sacrificing his time and his property in diffusing its blessings were fit subjects for the consideration of the British Parliament. After due deliberation, it was determined that his claims should be brought before the House of Commons by petition.

Previously, however, and in a degree preparatory to a direct application to the great national council, some of the chief personages in Gloucestershire began to take measures to give a testimony of the value in which he was held by those who knew him best and amongst whom his life had been spent. An advertisement appeared in the Gloucester newspapers expressive of these sentiments.

The late Earl of Berkeley took the lead in this expression of public feeling. If some were backward in seconding the Earl, Dr. Jenner's friends, exerted themselves with an energy which more than counterbalanced any discouragements they met with. The Countess of Berkeley also applied herself with her usual earnestness to effect the object. Her ladyship, assisted by Mr. Henry Hicks, the Rev. Mr. Pruen, and other personal friends of Dr. Jenner, had the satisfaction, in a short time, of seeing such a list of subscribers as enabled them to give an order for a small service of plate, which was executed by Messrs. Rundells and Bridge, with appropriate devices and the following inscription:—

Presented
by the
Nobility and Gentry
of the
County of Gloucester
to their Countryman
EDWARD JENNER, M.D., F.R.S.,
as a Testimony of the high sense they entertain
of
those eminent abilities which discovered,
and
that disinterested philanthropy which promulgated,
the Vaccine Inoculation.

Jenner's presence was again required in London; his claims for a national expression of gratitude were on the point of being submitted to Parliament. As the application ultimately referred to a money-grant it was necessary that the consent of his Majesty's ministers should be first obtained. Dr. Jenner had an interview with the premier on this subject, on the 12th of January, 1802. This conference was highly satisfactory. Unqualified approbation was expressed of the measure by the minister and he fixed upon an early day for the presentation of the petition. Mr. Addington spoke likewise strongly in praise of the discovery, and of the benefit that all the world was likely to derive from it. Much negotiation ensued with respect to the person who should present and second the petition to the House of Commons. The following letter upon the subject may not, perhaps, be uninteresting.

FROM W. WILBERFORCE, ESQ. TO DR. JENNER.

*Palace Yard, Feb. 24th,* 1802.

MY DEAR SIR,

I have often thought of addressing you on the subject we conversed about formerly, that I mean of your valuable discovery becoming the topic of parliamentary discussion, with a view to your receiving some compensation for your eminent services to the community. I hoped long ere now to see the matter brought forward. I always intended whenever it should be so to give you my best assistance on a principle of duty. I really thought, as I told you, that there were reasons why I was by no means an eligible introducer of the subject, and I could not just now undertake it, on account of my being engaged to render a similar service (though contrary to my own judgment) to another gentleman. But are you aware that Friday next is the last day for presenting private petitions, and that a petition is the proper mode of bringing your discovery before Parliament?

If I can be of any use in advising you I shall be unaffectedly glad, and in rendering you any assistance I am able.

At all events, I am persuaded you will do justice to the motive which prompts me to address you thus frankly, and believe me with esteem and regard, Dear Sir,

Your faithful servant,

W. WILBERFORCE.

It must be evident, we think, that this letter of Wilberforce was a cold one. How tame it is in comparison with what he wrote, and thought, and did in reference to Negro Slavery. But it often happens that philanthropic sympathies are most warmly interested in distant objects. Men subscribe for the relief of the Kamschatkans or the Hindoos, who will not patronize the hospitals or dispensaries in their own parish.

On the 17th of March, 1802, the following petition was presented.

To the Honourable the Commons of the United Kingdom of Great Britain and Ireland, in Parliament assembled.

The humble Petition of EDWARD JENNER, Doctor of Physic,

SHEWETH,

That your petitioner having discovered that a disease which occasionally exists in a particular form among cattle, known by the name of the cow-pox, admits of being inoculated on the human frame with the most perfect ease and safety, and is attended with the singularly beneficial effect of rendering through life the persons so inoculated perfectly secure from the infection of the small-pox.

That your petitioner after a most attentive and laborious investigation of the subject, setting aside considerations of private and personal advantage, and anxious to promote the safety and welfare of his countrymen and of mankind in general, did not wish to conceal the discovery he so made on the mode of conducting this new species of inoculation, but immediately disclosed the whole to the public; and by communication with medical men in all parts of this kingdom, and in foreign countries, sedulously endeavoured to spread the knowledge of his discovery and the benefit of his labours as widely as possible.

That in this latter respect the views and wishes of your petitioner have been completely fulfilled, for to his high gratification he has to say that this inoculation is in practice throughout a great proportion of the civilized world, and has in particular been productive of great advantage to these kingdoms, in consequence of its being introduced, under authority, into the army and navy.

That the said inoculation hath already checked the progress of the small-pox, and from its nature must finally annihilate that dreadful disorder.

That the series of experiments by which this discovery was developed and completed have not only occupied a considerable portion of your petitioner's life, and have not merely been a cause of great expense and anxiety to him, but have so interrupted him in the ordinary exercise of his profession as materially to abridge its pecuniary advantages, without their being counter-balanced by those derived from the new practice.

Your petitioner, therefore, with the full persuasion that he shall meet with that attention and indulgence of which this Honorable House may deem him worthy, humbly prays this Honorable House to take the premises into consideration, and to grant him such remuneration as to their wisdom shall seem meet.

The prime minister, Mr. Addington, (now Viscount Sidmouth) informed the House that he had taken the King's pleasure on the contents of the petition, and that His Majesty recommended it strongly to the consideration of Parliament. It was referred to a committee, of which Admiral Berkeley was appointed chairman, the inquiries of which were chiefly directed to these points:—first, the utility of the discovery itself; secondly, the right of the petitioner to claim the discovery; thirdly, the advantage in point of medical practice and pecuniary emolument which he has derived from it. The Committee went through a large body of evidence of various kinds and drawn from various sources. It was still confirmatory of Jenner's views, and highly flattering to him. But Dr. Pearson would appear to have attempted a second time to have robbed Jenner of the credit due to him. Dr. Baron says:—

"Of Dr. Pearson's statements before the Committee of the House it is by no means easy to give a lucid view. He sets out with asserting that he was *conversant* with the vaccine inoculation ever since January 1799, and as though he would bespeak credit for himself by exciting a partial sympathy for Dr. Jenner's claims, he thus proceeds: 'I think it but justice to Dr. Jenner to state that I am acquainted with the practice of inoculation of persons for the small-pox, who on good evidence have been said to have gone through the cow-pox since June or July 1798—the result of which was that they could not receive the small-pox infection.' He then goes on to say, in answer to questions put by the Committee, that his own knowledge of the vaccine inoculation was obtained by him '*in the first instance from Dr. Jenner*; afterwards I got *information* from other sources.' These sources have been already mentioned—and, with respect to the information derived from them, Dr. Pearson declares that he *imagines* that *it* and Dr. Jenner's first publication 'were independent of each other.' He next states that Mr. John Hunter did not mention in his lectures the *inoculation* of the cow-pox, but simply that persons who had had that disease could not take the small-pox, and that it had not been known to prove fatal in any one instance; that the Rev. Herman Drew did not lay claim to the discovery of inoculating with vaccine matter from one human being to another; that *that* discovery was exclusively Dr. Jenner's; that the events mentioned in the documents handed in took place earlier than 1798, because immediately on the publication of Dr. Jenner's Inquiry in that year he, Dr. Pearson, wrote to the gentlemen who furnished the information, and they '*immediately communicated their cases of vaccine inoculation* without appearing to be acquainted with Dr. Jenner's work." 499.

On the whole, the evidence of Dr. Pearson was intended to prove that vaccine inoculation had been practised by others before Dr. Jenner. His second examination before the Committee had a different object. It went to show that, though Dr. Jenner promulgated the practice of vaccination, he really knew very little about the matter; that his opinions as to its origin were erroneous; and that it required the experiments and labours of other observers to correct his mistakes: and that he and Dr. Woodville had the chief merit in the establishment of the Vaccine Inoculation. Thus, as Dr. Baron remarks, all that was left to Jenner was the merit of being the publisher of a provincial rumour, the nature of which he did not fully understand.

There cannot be a doubt that the behaviour of Dr. Pearson was of anything but a creditable character. We shall not go into the question, nor weigh in a nicely critical balance the exact quantum of merit on the part of Jenner. This is certain. He seized on a popular tradition—patiently investigated its truth—persisted through good and through bad report in submitting it to the test of experiment—dared to inoculate the human subject, and converted the imperfect, irregular, and precarious supply of vaccine lymph into an abundant and certain stock of it—and, neglecting all other things, devoted his time, attention, and abilities to the prosecution and perfection of this preventive of one of the scourges of our race. That such a person should from many receive discouragement and meet with ridicule while prosecuting his inquiry, and be treated with disparagement and robbed of his merits afterwards, is too true. Yet it is no less true than common. Such is, and ever has been, the fate of discoverers. Such was the lot of Newton, and this great man complained bitterly of these results of scientific discovery.

The committee reported favourably for Jenner, and the chairman moved that a sum not less than £10,000. be granted to him. Various opinions were expressed on that amount. Many thought £20,000. the least that could be given—a few even seemed to grudge the lesser sum. But, after various opinions had been offered, the Question was put that the words ten thousand pounds do stand part of the resolution; when the Committee divided,—

Ayes, 59. Noes, 56.—Majority, 3.

There cannot be a second opinion on the beggarly economy of this parliamentary grant. The very virtuous Chancellor of the Exchequer, who, good man, was alarmed for the pockets of the nation, pensioned off more handsomely many a titled whore, and refused to science that public treasure which was profusely squandered for the purposes of party. But it has always been thus in Britain. The factions which rule her, nearly balanced in power, and narrowly watched by each other, can ill afford to divert from their particular uses the loose cash of the people. A vote is of more consequence to them than any philosophical discovery, and medicine, which stands aloof from politics, and is not represented in legislative assemblies, receives little countenance from politicians. Religion, as a state instrument, obtains state support, but we suspect that were not her aid requisite in consolidating social order, even she would be left to shift for herself by the sordid spirit of rulers.

An attempt was made, or, rather, the idea was entertained, to raise a public subscription for Jenner. But nothing appears to have come of it. Jenner was addressed by the Medical Society of London, and by other medical societies, and elected an honorary associate by the Physical Society of Guy's Hospital. But these, though gratifying, were comparatively trivial circumstances. In the midst of all he still preserved his original simplicity and playfulness of character, and the following dialogue written about the time that he was most exposed to the vexatious opposition directed against him before the Committee of the House of Commons, is preserved by Dr. Baron, and may be quoted by us.

"DIALOGUE BETWEEN E. J. AND HIS SERVANT RICHARD.

R. A servant, Sir, has call'd just now,
And left a very handsome cow.
'Twas brought, he told me—let me see—
From some such place as *Hitaly*.
Well have I look'd her o'er and o'er,
And never saw her like before.
In no one point, Sir, does she fail,
Head, horn, neck, carcase, limbs or tail;
And here she is to take her station
In compliment to Vaccination.
J. Well, Richard, this is very good,
But how shall we contrive her food?
Instead of town, were we at home,
Among our meadows she might roam;
But here I've not one inch of pasture—
R. Oh, Sir, don't vex; that's no disaster,
The cow is only Paris Plaster!

*Bond Street*, 1802."

After the completion of the parliamentary inquiry, Jenner returned into the country, and hoped to enjoy repose with his family in his "pleasant home." But the "Royal Jennerian Society" was proposed, and organised in London—royal patrons were sought and found—Jenner was elected President—and on the 3rd of February, 1803, he took the President's seat at the London Coffee House. At subsequent meetings a central house for the institution, in Salisbury Square, was secured; a resident inoculator and medical secretary was appointed, and Dr. John Walker was elected to fill this situation.

On the 17th of May, 1803, his birth-day was celebrated by a public dinner. Jenner went with a deputation to thank His Majesty for his patronage—thirteen vaccine stations were opened in different parts of the metropolis—and all seemed to be going smilingly for the Royal Jennerian Institution. But the seeds of its destruction were sown in the election of Dr. John Walker. This individual ran counter to his instructions—opposed Jenner's views—contravened his practice—and endangered the cause of vaccination, while he made Jenner ridiculous. His removal was necessary, and, though difficult, was accomplished. But the schism had destroyed the concern, and on the appearance of the National Vaccine Institution, it disappeared.

In 1802, vaccination was communicated to the American Indians—in 1803, Jenner was elected an honorary life governor of the Humane Society, and, in October of the same year, Jenner received, among other poetical addresses, a Carmen Alcaïcum from Christopher Anstey, the witty author of the "Bath Guide." Dr. Baron quotes some stanzas from the Carmen which we think worth quoting again.

O! qui secundo natus Apolline
Incumbis arti pæoniæ, studens
Arcana Naturæ, gravemque
More novo prohibere morbum,

Jennere, laudes an sileam tuas?
Dum mente sanus, nec cythara carens,
Turpive succumbens senectæ
Rura vagor per amœna Cheltæ?

Furore quod non ante domabili
Tot dira Pestis quæ peperit mala,
In gentis humanæ levamen,
Te medico superata cessit;

Quippe arte mira quæ tibi contigit,
Puris benigni guttula, ab ubere
Inserta vaccino lacertis,
Corporeas penetrat meatus,

Brevique facta in vulnere pustula,
Propulsat Hostem, nec sinit amplius
Inferre morborum cohortes
Innumeras, comitemque mortem.

---

Te mater ambit filiolo cavens
Ut tuto ab atra corpore, sit lue,
Innupta te virgo decentes
Sint memori sine labe malæ?

Utcunque nostris laudibus invidens
Gens quæque grates dat tibi debitas;
Te Gallus extollit, tuamque
Obsequiosus adorat artem.

---

Nec longiori carmine te morer,
Mentemque curis utilioribus
Jennere seducam,—valeto.—
Teque, tuosque, precor, labores

Deus benigno numine prosperet;
Et dum perennis gloria Laureæ
Insignit Heroas Brittanos,
Civica te decorat Corona.

Jenner received diplomas and honours from abroad, pari passu with addresses and poems at home. Nay he was requested to intercede with the French authorities, to procure the liberation of some of the Détenus. He did intercede, and apparently, with partial success. Besides high honours paid to him in Spain, that country evinced an activity quite astounding. Who that knows any thing of Spanish phlegm, would imagine for an instant, that the Spanish monarch actually resolved to fit out an expedition for the express purpose of carrying to all the possessions of the crown of Spain beyond the seas, and to those of several other nations, the inestimable gift of vaccine inoculation. But the most surprising thing of all is, that the Spaniards not only thought of doing this but did it. We would venture to say that there is scarcely such another instance of decision to be found in the history of modern Spain. Thus in little more than six years after the promulgation of vaccination, it was known in every clime. Proceeding eastward and westward from our own island, it traversed the circumference of the globe. This fact is we think a sufficient refutation to the idea that vaccination met with great obstacles. The progress of truth is not so seriously nor so long impeded by bigotry as is imagined. It suits the views of unfortunate speculators and of wild theorists to say so. Finding opposition successful against themselves, they triumphantly appeal to equally potent opposition advanced against discoveries of paramount value. But they are in error when they appeal to the history of science. Unless religion has been mixed up with it, opposition to scientific truth has neither been very general nor successful. Certainly it was neither in the case of vaccination.

Inadequate as was the Parliamentary grant to Jenner, its actual results could hardly have been anticipated. The public thought that, as it had purchased, it had a right to make use of his services, and the applications to him for all kinds of information were endless.

"Influenced by the remarks of some of his parliamentary advocates, he was induced to fix himself in Hertford-street, May Fair. The result of this plan by no means corresponded with their anticipations. 'Elated and allured,' he observes, 'by the speech of the Chancellor of the Exchequer, I took a house in London for ten years, at a high rent, and furnished it; but my first year's practice convinced me of my own temerity and imprudence, and the falsity of the minister's prediction. My fees fell off both in number and value; for, extraordinary to tell, some of those families in which I had been employed, now sent to their own domestic surgeons or apothecaries to inoculate their children, alleging that they could not think of troubling Dr. Jenner about a thing executed so easily as vaccine inoculation. Others, who gave me such fees as I thought myself entitled to at the first inoculation, reduced them at the second, and sank them still lower at the third.'" 4.

He remarks to one of his correspondents :—

"I have now completely made up my mind respecting London. I have done with it, and have again commenced village-doctor. I found my purse not equal to the sinking of a thousand pounds annually (which has actually been the case for several successive years,) nor the gratitude of the public deserving such a sacrifice. How hard, after what I have done, the toils I have gone through, and the anxieties I have endured in obtaining for the world a greater gift than man ever bestowed on them before (excuse this burst of egotism), to be thrown by with a bare remuneration of my expenses!" 4.

In June, 1804, two years after the parliamentary grant, the Treasury still withheld the payment of it, and when, at last, it was actually made, he was mulcted of nearly £1,000 in the shape of official fees! A pretty mode of applying the national bounty. The Directors of the Vaccine Board felt this so sensibly, that a committee was appointed to inquire whether Dr. Jenner was not a sufferer in his income and pecuniary circumstances. That committee found that his emoluments from vaccine inoculation were not on an average more than £350 per annum, and there was clear proof that the gross deficit of capital in the four years immediately subsequent to his removal to London amounted to nearly £6,000.

It appears, at first sight, singular that this discovery should not have been a more fertile source of professional advantage to Jenner, yet, perhaps, the case admits of explanation. Jenner had devoted his whole attention to vaccination—had become, or at least was popularly considered, a vaccinator and nothing else—and was not likely to be employed for other things in London, where reputation for particular things brings usually a particular kind of practice. Vaccination itself was apparently so simple, so easily performed by any one, that the regular attendants on a family could, or at all events would perform it, at a cheaper rate than Jenner himself. Jenner, indeed, quoad vaccination, was on the horns of a dilemma. To procure its universal adoption, he must show its simplicity, its trivial character—to do this was to dispense with the necessity for his personal services. Still, with all allowance for circumstances of this nature, Jenner could not have been "cut out" for London practice, or he would have made more of it than he did.

He was much annoyed with the virulent opposition excited against him by the bigotry of the opponents of vaccination on the one hand, and by the envy of some of its supporters on the other. Reports of failures were industriously spread, and pamphlets were even published. Yet there were honours in the opposite scale. The Medical Society of London voted him a gold medal—the corporations of Dublin and Edinburgh the freedom of their cities—one of the Napoleon series of medals was struck in commemoration of vaccination—and a direct application to the Emperor met with flattering success. The application in question was this :—Dr. Wickham, one of the travelling fellows of the University of Oxford, was at Paris in the first years of his travels, when the command for the detention of the English was issued. He was permitted to retire to Geneva on his parole. Another young Englishman of the name of Williams, was also detained. The situation of both these gentlemen had been particularly submitted to the consideration of Dr. Jenner : that of Dr. Wickham presented peculiar claims. Having, in vain, addressed the Central Committee of Vaccination in Paris, Jenner appealed in the following letter to the Emperor himself.

" Sire,—Having by the blessing of Providence made a discovery of which all nations acknowledge the beneficial effects, I presume upon that plea alone, with great deference, to request a favour from your Imperial Majesty, who early appreciated the importance of vaccination and encouraged its propagation, and who is universally admitted to be the patron of the arts.

My humble request is that your Imperial Majesty will graciously permit two of my friends, both men of science and literature, to return to England ; one, Mr. William Thomas Williams, residing at Nancy; the other, Dr. Wickham, at present at Geneva. Should your Imperial Majesty be pleased to listen to the prayer of my petition, you will impress my mind with sentiments of gratitude never to be effaced.

I have the honour to be, with the most profound deference and respect,

Your Imperial Majesty's

Most obedient and humble servant,

E. J."

It was on this, says Dr. Baron, or on some similar occasion, when Napoleon was about to reject the proffered petition, that Josephine uttered the name of Jenner. The Emperor paused for an instant, and exclaimed, "Jenner! ah, we can refuse nothing to that man." Early in July, 1806, Mr. Williams received an intimation that the Emperor had listened to Dr. Jenner's petition, and had granted liberty both to himself and Dr. Wickham. A high compliment, this fact, both to Jenner and the Emperor. The latter, indeed, was in many respects a generous despot. His was not the cold tyranny of a mere military chief. A profound historian has remarked that those best excite enthusiasm who feel it, and the devotion of the nation to Napoleon was the consequence of numerous acts like these. Even despotism is loved in the genius and generosity of the despot.

Dr. Baron adverts to the report that was circulated, and re-circulated so late as 1827, in reference to Jenner's inoculating his youngest son with small-pox. This was held up as a commentary on his advocacy of vaccination. It is right to dispose of this injurious rumour, discreditable, if substantially true, to Jenner's head or heart. The real facts were the following. They are contained in the latter part of a long letter of Jenner's.

"My two eldest children," he says, "were inoculated for the small-pox before I began to inoculate for the cow-pox. My youngest child was born about the time my experiments commenced, and was among the earliest I ever vaccinated. By referring to the first work I published on the subject in the spring of the year 1798, page 40, you will find his name, Robert F. Jenner, and you will observe it noticed, that on his arm the vaccine lymph did not prove infectious. It advanced two or three days, and then died away. In a short time after I was necessitated to go with my family to Cheltenham for a few months, where I did not think it prudent to resume my operations, from a supposition that the people assembled at a public watering-place might conceive the disease (then so little known) to be contagious, and that it might excite a clamour. However, during my stay there, this boy was accidentally exposed to the small-pox, and in such a way as to leave no doubt on my mind of his being infected. Having at this time no vaccine matter in my possession, there was no alternative but his immediate inoculation, which was done by Mr. Cother, a surgeon of this place, who is since dead; but this history is well known to many who are living.

You now see on what a baseless foundation the insinuations which have been published respecting these facts rest." 49.

Vaccination made rapid progress abroad, and, perhaps, in spite of obstacles, opposition, and envy, not very unsatisfactory advance at home. But the state of his private fortune occupied the attention both of Jenner and his friends. Fortunately, admiration for the discoverer of vaccination made Lord Henry Petty one of the number, and a friend of no contemptible description. On the death of Mr. Pitt, his Lordship became Chancellor of the Exchequer, and, on the 2d of July, 1806, he brought the subject of vaccination once more before the House of Commons. The small-pox was again becoming prevalent, and the Chancellor proposed that an address to His Majesty should be voted by the House, praying "that his Royal College of Physicians be requested to inquire into the progress of vaccine inoculation, and to assign the causes of its success having been retarded throughout the United Kingdom, in order that their report may be made to this House of Parliament; and that we may take the most proper means of publishing it to the inhabitants at large."

This motion was carried unanimously, and the College of Physicians applied themselves diligently to the business referred to them. They collected evidence from all quarters, friendly and hostile, and the following is their temperate, and just Report—they strongly recommended the practice of vaccination, this conclusion being formed from an irresistible weight of evidence which had been laid before them; adding, that "when the number, the respectability, the disinterestedness, and the extensive experience of its advocates are compared with the feeble and imperfect testimonies of its few opposers; and when it is considered that many who were once adverse to vaccination have been convinced by farther trials, and are now to be ranked among its warmest supporters, the truth seems to be established as firmly as the nature of such a question admits; so that the College of Physicians conceive that the public may reasonably look forward with some degree of hope to the time when all opposition shall cease, and the general concurrence of mankind shall at length be able to put an end to the ravages at least, if not to the existence, of small-pox.

On the 29th of July, 1807, the Chancellor of the Exchequer, the late Right Honourable Spencer Perceval, moved the order of the day for the house to go into a committee of supply; and stated that it was referred to that committee to consider of a farther sum to be allowed to Dr. Edward Jenner for the discovery of the vaccine inoculation, and his communication of it to the world. The house resolved itself into a committee accordingly; the late Sir Benjamin Hobhouse being in the chair.

The debate which arose on the motion of the Chancellor of the Exchequer was important; and evinced, with one or two exceptions, a proper estimate of the nature of the question. The excellent report of the College of Physicians afforded the ground on which the Chancellor of the Exchequer proposed "that a sum not exceeding £10,000 be granted to His Majesty, to be paid to Dr. Edward Jenner, as a reward for promulgating his discovery of vaccine inoculation; and that the same be issued without any fee or other reward whatever."

Mr. Shaw Lefevre represented the anti-vaccinists. But he represented it alone. Mr. Edward Morris took a different view of the subject. Influenced entirely by the weight of evidence, he made a powerful appeal to the house; and concluded by saying, "after what I have seen and heard, and know to have been proved upon this subject, I feel myself called upon to move that instead of £10,000, £20,000 be inserted in this resolution." This was warmly supported by many, countenanced by more; and the question was put that £20,000 do stand part of the resolution: when the committee divided; ayes, 60; noes, 47; majority, 13.

Thus, at last, something like a moderate recompense was awarded by the representatives of the nation to Jenner. But it was not during the reign of Mr. Pitt. That extraordinary man husbanded the resources of the country in detail that he might squander them in gross; and his treatment of Dr. William Hunter and of Jenner are proofs amongst others of the comparative indifference he evinced towards science. Had he lived in peaceful times it might, perhaps, have been otherwise.

In 1806, the Spanish expedition returned, having circumnavigated the globe, and carried to the regions of the rising and the setting sun the benefits of vaccination. And if, as has been said, Godoy, was the author of that philanthropic voyage, that alone should clear him with posterity.

From Bengal Jenner received a subscription of four thousand pounds, raised in honour of his discovery and himself—from Bombay, two thousand pounds—and from Madras, one thousand three hundred and eighty-three pounds.

Among the ardent advocates of vaccination, the reverend and eccentric Rowland Hill was foremost. He vaccinated and preached, and preached and vaccinated. He once introduced Jenner to a nobleman in these terms:—"allow me to present to your Lordship my friend Dr. Jenner, who has been the means of saving more lives than any other man."—"Ah! would I, like you, could say *souls*."

The native tribes of North America received the gift of vaccination, and expressed their gratitude in accordance with their customs to the donor. They sent him a belt and string of Wampum, taught their children to speak his name, and solemnly commended him to the Great Spirit. And if that "Great Spirit," as no doubt He does, befriends the benevolent and good, the prayer of those simple Indians has been heard, and, "in the land of Spirits beyond the waters," the discoverer of vaccination tastes a reward, which neither parliaments can vote nor nations bestow.

It appears that at Ringwood, in Hampshire, vaccination *appeared* to have been in some measure unsuccessful. Under these circumstances, a deputation of the Royal Jennerian Society went down to inquire into the facts. It is stated that the deputies carried pistols to defend themselves against the populace! In reference to this, Dr. Baron observes:—

"The force of prejudice and error, and the evil consequences which have resulted from them, form the most melancholy chapters in the history of man; at one time struggling to maintain false and inaccurate dogmas, at another resisting the plainest and most convincing demonstrations, we are compelled to believe that there is a principle in our nature which has too strong an affinity for what is untrue, to permit the understanding either to discern or acknowledge an opposite principle, till both the moral and intellectual vision have been purified and strengthened. The persecutors of Galileo would, I believe, have been eclipsed in their monstrous and outrageous hostility to the splendid discoveries of that illustrious man, by some of the opponents of vaccination, had the spirit of the age or their own power enabled them to carry their wishes into execution. It is very true that the persons who manifested such dispositions were little distinguished by their rank, or station, or abilities. They were, nevertheless, men of education, and many of them belonged to the medical profession; and I record it as a striking proof of the weakness of the human understanding, that in the nineteenth century, and in the metropolis of the British Empire, two of the most beneficial inventions should, in protracted and repeated public discussions, have been consigned to contempt and obloquy, and their authors held up to the world as hypocrites and impostors. On Monday the 28th of March, 1808, the following question was discussed at the British Forum:—'Which has proved a more striking instance of the public credulity,—the gas lights of Mr. Winsor, or the cow-pox inoculation?' The result of the discussions was as usual announced; and both vaccination and gas lights were handed over to scorn and ignominy!" 111.

We apprehend that Dr. Baron is wrong in supposing that there is an innate love of falsehood in the human mind. Cæteris paribus there is a love of truth. Exhibitions of the nature alluded to by Dr. Baron do not prove his position. They exhibit the force of interest and prejudice.

We turn from these contemptible exhibitions of weakness or ill-nature, to some of a more gratifying character. A young man of the name of Powell, the son of W. D. Powell, Esq. Chief Justice of the Court of King's Bench in Upper Canada, was captured on board of Miranda's squadron by the forces of the King of Spain; tried, and sentenced to ten years confinement and hard labour. To obtain his release, Dr. Jenner memorialized the King of Spain, and, to the honour of both, the appeal was successful. The Emperor of Austria granted a similar boon. Napoleon was again addressed, and again evinced his respect for science in the person of Jenner. The Baron Corvisart writes to the latter:—

"J'ai remis, ces jours derniers," (he observes, in a letter dated Paris, Dec. 5, 1809), "à S. M. la copie de vôtre dernière lettre en date du 4 8bre 1809; L'Empereur m'a permis de vous repondere, Monsieur, qu'il ferait mettre en liberté les deux gentilhommes (MM. Garland et Gold), auxquels vous vous interessez. Je suis bien flatté de pouvoir vous annoncer cette heureuse nouvelle."

"The Baron," says our author, "then asks Jenner to render a good office to a young friend of his, a prisoner, who had been sent back to France on his parole. His best efforts were, I believe, exerted in behalf of the young man; but unhappily, Jenner's influence with the British government was not equal to that which he enjoyed with the court of France."

The haughty spirit of aristocracy is shewn to the life in this incident; and the whole of Jenner's career is an apt commentary on the text, that, "no man is a prophet in his own country." But Jenner was little less than a prophet abroad. For at a time when war was at its hottest, and personal animosities, as well as opinions, combined to render it rancorous, his certificate was given and received as a sort of passport, and availed more than the seals of the Foreign Office. The following is a certificate of this description:—

"I hereby certify, that Mr. A. the young gentleman who is the bearer of this, and who is about to sail from the port of Bristol on board the Adventure, Captain Vesey, for the island of Madeira, has no other object in view than the recovery of his health.

Edward Jenner,
Member of the N. I. of France, &c. &c.

*Berkeley, Gloucestershire July* 1, 1810."

If there is a science which deserves well of all men it is our's, since it is intended for the indiscriminate benefit of all. The feeling that it is an universal science should be fostered, and, individually and collectively, we should take all opportunities of extending to each other a reciprocity of good offices. We record these facts connected with Jenner with pleasure, because they place the conduct of some of the continental rulers in a favourable light, and because they give encouragement to that spirit of cosmopolitism which should prevail in all that relates to us.

In the early part of 1809, Government founded the National Vaccine Institution. Jenner was appointed director. But the name was chosen on the lucus à non lucendo principle, for the director had no power of directing. We shall not follow Dr. Baron through his analysis of the birth and first acts of this Institution. They were so painful to Jenner that he resigned his office, and, so far as we can see from the evidence adduced by Dr. Baron, he was right in doing so. But we quit this subject to pursue the course of Jenner's life.

In the Summer of 1808, his biographer made his acquaintance at Fladong's Hotel, in Oxford Street. Jenner was dressed in a blue coat, white waistcoat, nankeen breeches, and white stockings. All the tables in his apartment were covered with letters and papers on the subject of vaccination, and the establishment of the National Vaccine Institution. But the blue coat and white waistcoat were soon to be exchanged for garments of a more sombre hue, for his eldest son, Edward, sank into a consumption, and fell a victim to it in the beginning of 1809. The young man had been always delicate, and had evinced rather a defective understanding. Yet his father was deeply affected by his loss.

His health indeed was seriously impaired by the infliction, and change of air, as well as active remedies, were necessary. But his mind was as intent as ever on its darling theme, the diffusion of the benefits of vaccination. The practice of inoculation still prevailed, and many private as well as some legislative attempts were made to put it down. But, in this free country, such attempts are powerless in opposition to popular intelligence or prejudice. This is undoubtedly an evil. But it is an evil inseparable from freedom, and is as nothing when compared with *its* thousand benefits. If force could on every occasion be resorted to for the purpose of controlling the national will, it requires but little knowledge of governments to be certain, that such force would not be reserved for legitimate occasions. The best mode of removing error is to enlighten men, not to sabre or incarcerate them; and, in free countries, where discussion is unshackled, truth is certain to prevail at last.

To return to Jenner. His applications for the release of Englishmen confined by Napoleon had been favourably received by the Emperor through the representations of Corvisart and Husson, his physicians. The latter had a brother, Captain Husson, a prisoner of war in England. Jenner petitioned our Government for his release, and was refused. Another pregnant instance of the absence of generous sentiments, in the party governments of this country. Can we wonder at the sentiments entertained by foreigners? But they ought to know, and we trust they do, that the bigotry of the vulgar, of high rank or of low, is repudiated by the men of science in this country, and that acts of this description must be saddled on their proper authors.

In 1808, the National Institute of France elected Jenner a corresponding member, and, in 1811, that distinguished body placed him in the list of foreign associates. The choice was ratified by Napoleon.

In the year 1813, the question was brought before the University of Oxford, and in full convocation the degree of M.D. by diploma was unanimously voted to him. This proceeding, not less honourable to that distinguished body than to the individual who was thus signalised, it was imagined would open the portals of the College of Physicians to him, and remove all objections to his taking a seat at the vaccine board; but it will appear in the sequel that it turned out otherwise, for it was decided in that learned body that Jenner could not be made a Fellow unless he went through the usual examination. Well might Dr. Baillie exclaim against this with more than usual warmth. It was too ridiculous.

In 1814, Napoleon abdicated. The Bourbons were restored, and, in that year, the allied sovereigns visited England. Jenner went to London on that occasion, for the last time. He was presented to the Sovereigns, who all evinced an anxiety to see, none to reward him. Ribands and pensions were showered on those who had destroyed men—some honied words on him who had preserved them. Jenner's own account of his interview with the Emperor of Russia is characteristic.

"I was very graciously received, and was probably the first man who had ever dared to contradict the autocrat. He said, 'Dr. Jenner, your feelings must be delightful. The consciousness of having so much benefited your race must be a never-failing source of pleasure, and I am happy to think that you have received the thanks, the applause, and the gratitude of the world.' I replied to his Majesty that my feelings were such as he described, and that I had received the thanks and the applause, but not the gratitude, of the world. His face flushed; he said no more, but my daring seemed to give displeasure. In a short time, however, he forgot it, and gave me a trait of character which shewed both great goodness of heart and knowledge of human nature. My inquiries respecting lymphatic diseases, and tubercles, and pulmonary consumption had reached the ears of the Grand Duchess. She was present, and requested me to detail to her brother, the Emperor, what I had formerly said to her Imperial Highness. In the course of my remarks I became embarrassed. She observed this, and so did the Emperor. 'Dr. Jenner,' said she, 'you do not tell my brother what you have to say so accurately as you told me.' I excused myself by saying that I was not accustomed to speak in such a presence. His Majesty grasped me by the hand, and held me for some time, not quitting me till my confidence was restored by this warm-hearted and kind expression of his consideration." 207.

A few such cheap rewards of Princes was all that Jenner got.

On the 14th of September, 1815, Jenner lost his wife. He felt this loss acutely, and not only acutely but permanently. He went immediately to Berkeley, and never, but for a day or two, left it again. He had finally quitted public life, but he could not retire from it altogether. Vaccination was an affair of nations, public attention was ever open to it, public discussions were always keen upon it, and the noise of dispute could not fail to penetrate even to Berkeley.

In 1818, a virulent epidemic small-pox prevailed in many parts of Great Britain, as well as on the Continent. Many of the vaccinated contracted the disease, and then, of course, arose the usual flood of rumours, and apprehensions, and exaggerated stories of disasters. It was said, too, that the vaccine lymph had deteriorated, and thus, says Dr. Baron, there was an accumulation of evils and misrepresentations, which could not fail to annoy Jenner. Statistical facts, the gross amounts of the experience of the world, have, after forty years, established the benefits of vaccination. They may not be *quite* so great as its discoverer supposed they would be, but, if life and health and beauty merit the title of human blessings, then vaccination is the greatest boon that one man has ever conferred upon his species.

It may not be uninteresting to quote the last recorded opinions of Jenner. They were written a few days before his death, on the back of a letter, the post mark on which gives the date, January 14th, 1823. They express his deliberate sentiments at a time when age had mellowed sanguine anticipations, when ambition had received all that it could expect, when death had robbed him of those nearest and dearest to him, and when retirement and the consciousness of the approach of another scene conferred that sincerity and solemnity on parting words, which has often lent them a prophetic character.

"MY OPINION OF VACCINATION IS PRECISELY AS IT WAS WHEN I FIRST PROMULGATED THE DISCOVERY. IT IS NOT IN THE LEAST STRENGTHENED BY ANY EVENT THAT HAS HAPPENED, FOR IT COULD GAIN NO STRENGTH; IT IS NOT IN THE LEAST WEAKENED, FOR IF THE FAILURES YOU SPEAK OF HAD NOT HAPPENED, THE TRUTH OF MY ASSERTIONS RESPECTING THOSE COINCIDENCES WHICH OCCASION THEM WOULD NOT HAVE BEEN MADE OUT."

Jenner's last published work made its appearance in 1822. It was entitled, "A Letter to Charles Henry Parry, M.D., &c. on the Influence of Artificial Eruptions in certain Diseases Incidental to the Human Body." Not many months after the publication of this paper, Dr. Parry died. Jenner, as one of his oldest and most attached friends, went to Bath to attend his funeral. His own was not far distant. Before we bring his career to a close, we may imitate his biographer, and take a narrower look at the man, in his social character and domestic habits.

Dr. Baron assures us that the calmness and composure of Jenner amidst the irritating circumstances that surrounded him, were greatly owing to the influence and example of his wife—an excellent woman, of unaffected piety, good sense, and genuine amiability.

Jenner repaid the exertion of this influence by anxious tenderness and delicate attentions. For years before her death, Mrs. Jenner was chiefly confined to her own apartments. The manner in which Jenner superintended the arrangement of every thing that could be thought of for her comfort, the administration of her medicine and the preparation of her food, (which a difficulty of deglutition rendered necessary,) all indicated the warmest attachment and the kindest feelings.

Jenner was devoutly conscious of the omnipresence of the Deity, and believed that this great truth was too much overlooked in our systems of education.

His personal appearance to a stranger at first sight was not very striking; but it was impossible to observe him, even for a few moments, without discovering those peculiarities which distinguished him from all others. This individuality became more remarkable the more he was known; and all the friends who watched him longest, and have seen most of his mind and of his conduct, with one voice declare, that there was a something about him which they never witnessed in any other man. The first things that a stranger would remark were the gentleness, the simplicity, the artlessness of his manner. There was a total absence of all ostentation or display; so much so, that in the ordinary intercourse of society he appeared as a person who had no claims to notice.

If the representation of the bust of Jenner, prefixed to the present volumes, be a true one of the man, his countenance certainly would not strike the observer as one indicative of genius. His inquiries were necessarily conducted in a desultory manner. But broken as they seemed to be, one object was ever present, and it was only the mode of investigation that was vague. This was partly the result of circumstances, but partly, also, of original constitution of mind, and of the want of a very exact training of it. Dr. Baron, for example, informs us, that:—

" It ought at the same time to be mentioned, that neither Dr. Jenner's previous education nor his habits gave him a relish for any of the branches of pure science. He seemed to have a peculiar horror of arithmetical questions. He was often jocular on this defect in his nature; and I believe he frequently paid severely for it; as he would rather attend to any thing than pounds, shillings, and pence. A neighbour was once expending a great many words to draw his attention to some affairs of this kind. He expressed himself perfectly satisfied; but not so his neighbour. He continued to dwell upon the different items till Jenner's patience became exhausted; and he exclaimed that he would rather look for an hour at a mite through a microscope than have his time taken up with such things." 294.

It would be curious to sum up the names of great men who have been unable to comprehend the more exact parts of education. Many who have adorned, and some who have greatly benefited humanity, have been in this predicament—a proof that there are other roads to truth besides the high ways and direct ones.

The subjects of his studies generally lay scattered around him; and, as he used often to say himself, seemingly in chaotic confusion. Fossils, and other specimens of natural history, anatomical preparations, books, papers, letters—all presented themselves in strange disorder; but every article bore the impress of the genius that presided there. The fossils were marked by small pieces of paper pasted on them, having their names and the places where they were found inscribed in his own plain and distinct hand-writing. His materials for thought and conversation were thus constantly before him; and a visitor, on entering his apartment, would find in abundance traces of all his private occupations. He seemed to have no secrets of any kind; and, notwithstanding a long experience with the world, he acted to the last as if all mankind were trustworthy, and free from selfishness as himself.

In prosecuting his investigations into unexplored regions, analogy was his favourite guide. This method is characteristic of all original minds; and although it is often carried too far, it has been, when duly and cautiously followed, the parent of some of the greatest inventions. To it we are, in great degree, indebted for the discovery of the properties of the variolæ vaccinæ. But his analogies were sometimes hurried forward on the wings of imagination; and, of course, were not always accurate or conclusive. His language, too, on scientific subjects, though for the most part remarkably simple and precise, was, on some occasions, of too figurative a cast. This rich and flowery garb often seemed to overlay sterling treasure, and by those who could not penetrate below the surface he has been deemed rather visionary.

An analogical mind is one of quick perceptions. Wit is itself but a ready perception of analogy, and minds of this description are naturally best adapted for original discovery. Between minds of this sort, and those of an exact character, a sort of war has been always waged. And yet the world has benefited almost equally by both. It is the combination of the two, as in the case of Newton, that constitutes the maximum of human intellect. That great man wrote poetry and a hypothesis on the book of Revelations, at the very time that his mind was pregnant with the most severe calculations. And such a mind was precisely the one adapted for the discovery of fluxions and the system of the universe.

The habits of Jenner were perfectly simple, and the formality and reserve of society were foreign to him.

"In his latter years he was not a very early riser; but he always spent some part of his time in his study before he appeared at the breakfast table. When in London and at Cheltenham, he generally assembled his scientific and literary friends around him at this hour. Some came for the pleasure of his conversation; some to receive instruction in the history and practice of vaccination. In the country, where his guests were generally his own immediate connexions or his intimate friends, the originality of his character came out in the most engaging manner. He almost always brought some intellectual offering to the morning repast. A new fact in natural history, a fossil, or some of the results of his meditations, supplied materials for conversation; but, in default of these, he would produce an epigram, or a fugitive jeu d'esprit; and did not disdain even a pun when it came in his way. His mirth and gaiety, except when under the pressure of domestic calamity or bodily illness, never long forsook him; and even in his old age, the facility with which he adapted his conversation and his manners to the most juvenile of his associates was truly interesting." 291.

Jenner's mind was an active one. He seldom or never passed a butcher's shop without a peep at its contents; because he often found something to illustrate his views of comparative anatomy and pathology. He generally carried a large pocket-book with him; and recorded his thoughts as they occurred. His mind indeed was of a very inquisitive cast. He was ever starting some subject for consideration. Dr. Baron has often known him dictate to his young friends problems in physiology, pathology, or natural history for their investigation; and Dr. Baron candidly owns that some of the pathological questions which he has himself discussed, originated in this way, and were prosecuted with his help. He felt as all men of genius have done for the hardships of his less successful brethren. He used to say, laughingly, that medical men should follow the example of tradesmen, and bring their employers to a sense of justice by "a general strike."

It has been already seen that, notwithstanding the personal influence that Dr. Jenner had with foreign states, he had next to none at home. He never succeeded in procuring an appointment for any of his relatives or friends. He mentioned that all his attempts to get a living for his nephew George had failed, though addressed to quarters where they might, without presumption, have been expected to have met with attention and success.

But the time was at hand, when the emptiness of success, and the bitterness of disappointment would be alike disregarded.

On the 6th of August, 1820, he was walking in the garden, and became suddenly faint and giddy; he sank to the ground, and his hat dropped off. How long he remained in this state could not be ascertained, as he contrived ultimately to get into the house, where he was found in a somewhat confused state; and his clothes covered with earth. From this attack he gradually recovered, but he became remarkably sensitive to external impressions, particularly to sharp sounds, like the click of a knife upon a plate. He felt as if an electric shock shot through his frame. It cannot be a matter of surprize that he occasionally expressed impatience.

In the evening of the 24th of January, 1823, Jenner visited an old schoolfellow and friend, who after exposure to severe cold became apoplectic. Next morning, he arose as usual, was informed that his friend was dead, and seems to have made a memorandum of his case. He went down stairs to his library.

"As he did not appear at breakfast, the servant was sent to ascertain the cause. On entering the room, he found that he had sunk from the couch on the floor; and that he was lying in a state of insensibility. His nephew, Mr. Henry Jenner, and Mr. Henry Shrapnell, were with him in a few minutes, and administered the most judicious remedies. A messenger was sent for me; and I reached Berkeley about two in the afternoon. I found him in bed, lying in a complete state of apoplexy. The right side was paralysed; the pupils of the eyes contracted to a point, and unaffected by strong light; the breathing stertorous, with a general insensibility to almost every external impression. Every effort was employed to arouse him from this condition; but the fatal character of the malady became more and more apparent, and he expired about three o'clock in the morning." 314.

Thus died Edward Jenner. He was buried at Berkeley by the side of his wife. A summary of his character appears unnecessary, for every page of his life has shewn him in the same light—a good, and in many respects a great man. His epitaph, like Wren's, may be safely written :—

"Si velis monumentum, circumspice."

The combination of moral worth and intellectual eminence is too valuable as an example to be lost sight of. All who wish well to their species should insist upon it, and hold it up for veneration. We do not regret the space we have devoted to the life of Dr. Jenner.

